# Functional Roles of the Complement Immune System in Cardiac Inflammation and Hypertrophy

**DOI:** 10.3390/ijms26209931

**Published:** 2025-10-12

**Authors:** Kathryn D. Hok, Haydn E. Rich, Anthony Shadid, Lavanya Gunamalai, Tingting Weng-Mills, Rajarajan A. Thandavarayan, Nirmal K. Banda, Marie-Francoise Doursout, Marcos I. Restrepo, Pooja Shivshankar

**Affiliations:** 1 Center for Metabolic and Degenerative Diseases, The Brown Foundation Institute of Molecular Medicine for Prevention of Human Diseases, UTHealth-McGovern Medical School, Houston, TX 77030, USA; kathryn.d.hok@uth.tmc.edu (K.D.H.); haydn.e.rich@uth.tmc.edu (H.E.R.); anthony.shadid@bcm.edu (A.S.); 2 Center for Human Genetics, The Brown Foundation Institute of Molecular Medicine for Prevention of Human Diseases, UTHealth-McGovern Medical School, Houston, TX 77030, USA; lavanya.gunamalai@uth.tmc.edu; 3 Department of Biochemistry and Molecular Biology, UTHealth-McGovern Medical School, Houston, TX 77030, USA; tingting.weng@uth.tmc.edu; 4 Department of Cardiology, DeBakey Heart & Vascular Center, Houston Methodist Weill Cornell Medical College, Houston, TX 77030, USA; ramirthalingamthandavarayan@houstonmethodist.org; 5 Division of Rheumatology-Department of Medicine, School of Medicine, University of Colorado Anschutz Medical Campus, Aurora, CO 80045, USA; nirmal.banda@cuanschutz.edu; 6 Department of Anesthesiology, UTHealth-McGovern Medical School, Houston, TX 77030, USA; marie-francoise.doursout@uth.tmc.edu; 7 VA-San Antonio Geriatric Research Education and Clinical Center (GRECC)-South Texas Veterans Health Care System Audie L. Murphy Division, UTHealth San Antonio, San Antonio, TX 78229, USA; restrepom@uthscsa.edu

**Keywords:** complement immune system, complement anaphylatoxins, cardiac inflammation, cardiac hypertrophy, cardiac remodeling, autoimmune diseases, cardiovascular events, infectious diseases, genetic defects in complement genes, complement in metabolic diseases, cardiovascular adversities, cardiovascular diseases, cardiac arrhythmias

## Abstract

Cardiac inflammation and hypertrophy develop as a pathologic response to an array of insults, such as myocardial infarctions, chronic systemic hypertension, and valvular defects. Due to the high prevalence of such conditions, there is an increasing need to prevent and halt cardiac hypertrophy. Because cardiac damage and subsequent remodeling can lead to arrhythmias, heart failure, and even sudden cardiac death, inhibition of cardiac hypertrophy is key to reducing cardiovascular-related mortality. The immune system is the driving force behind inflammatory reactions. All three pathways of complement system activation—classical, lectin, and alternative—are implicated in developing cardiac damage, inflammation, and hypertrophy due to infectious and non-infectious causes, autoimmune diseases, genetic polymorphisms, and forms of complement dysregulation. Of interest in this review is the role of the complement system, a collection of soluble and membrane-bound proteins that mediate inflammatory processes through interactions with signaling molecules and immune cells. This review comprehensively discusses the roles of these complement pathways in contagious, chronic inflammatory, genetic, and metabolic diseases. An overview of the completed and terminated clinical trials aimed at preventing cardiovascular mortality by targeting various aspects of the complement system and inflammatory reaction is included. Most current treatments for cardiac inflammation and remodeling primarily target the renin–angiotensin–aldosterone system (RAAS), which prevents further remodeling by reducing myocardial workload. However, moving forward, there may be a place for emerging anti-complement therapeutics, which impair the inflammatory response that generates hypertrophy itself.

## 1. Introduction

As a significant part of the innate immune response, the complement system has widespread involvement in autoimmune and infectious diseases. It has also been indicated in an array of other inflammatory pathways. This review article notes the complement-induced pathogenesis of cardiac inflammation and hypertrophy. Cardiac hypertrophy can be described as either physiological or pathological, in which the physiological course occurs as a normal response to a non-disease-related stimulus, and pathological hypertrophy is a maladaptive response to a disease-related stimulus [1]. For instance, physiologic hypertrophy may occur during pregnancy, an everyday biological occurrence, while pathologic hypertrophy is caused by a chronic hypertensive state as part of a disease process [1]. Chief among the differences between these two processes is that physiologic hypertrophy is primarily considered reversible.

**Figure 1:**
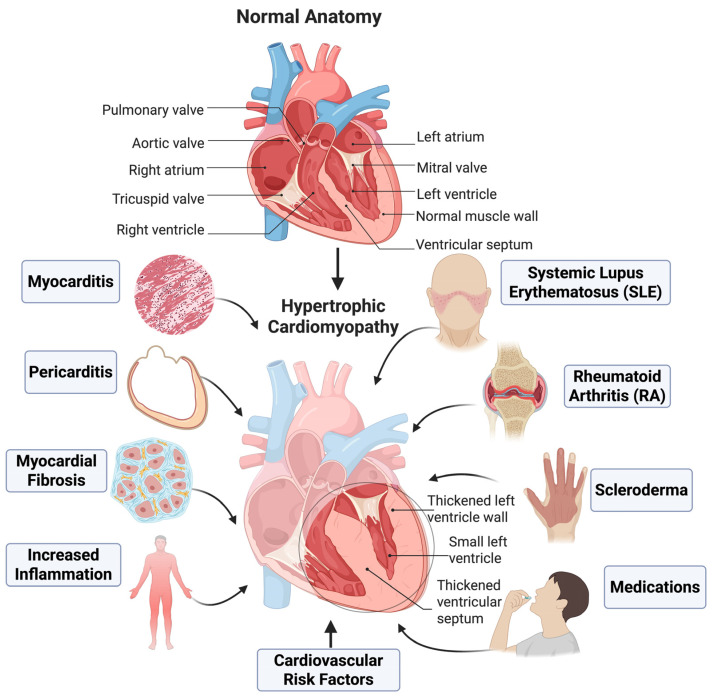
Chronic inflammatory and autoimmune conditions contribute to the development of hypertrophic cardiomyopathy (HCM). Inflammation plays a significant role in various cardiovascular diseases, including cardiomyopathies. It can activate molecular pathways that contribute to cardiomyocyte hypertrophy, dysfunction, and fibrosis. Chronic inflammation, including myocarditis, pericarditis, and fibrosis in the myocardium, can lead to cardiac hypertrophy and dysfunction, which may cause complications like arrhythmias, heart failure, and sudden cardiac death. Autoimmune conditions like rheumatoid arthritis (RA) and systemic lupus erythematosus (SLE) have been associated with HCM. Studies have documented cases where HCM coexists with RA. Scleroderma, or systemic sclerosis, is an autoimmune disease characterized by fibrosis of the skin and internal organs, including the heart. Certain medications can also induce or exacerbate cardiomyopathy, including those that can cause cardiac hypertrophy.

In contrast, pathologic hypertrophy sees a measure of fibrosis that later may contribute to cardiac dysfunction [2]. This review focuses on the role of complement activation and regulation in developing pathological cardiac inflammation and hypertrophy. It explores how the complement system, a crucial part of the innate immune response, contributes to the progression of heart diseases characterized by increased heart muscle mass and inflammation. An overview of chronic inflammatory and autoimmune conditions that contribute to hypertrophic cardiomyopathy is illustrated in Figure 1.

Pathologic cardiac hypertrophy refers to an enlargement of the heart in response to increased pressure or volume of systemic blood circulation, a phenomenon often described as hemodynamic overload [1]. In the new state of hypertrophy, the heart exerts increased contractility against the increased opposing forces. There are two types of cardiac hypertrophy, depending on the stressor the heart is adapting to. The first is known as concentric hypertrophy, which describes the growth of sarcomeres, or muscle fibers, in thickness, increasing the width of the cardiac wall. This form of hypertrophy is seen in response to pressure overload, such as valvular stenosis. The second type of hypertrophy is described as eccentric, in which myocytes are lengthened. Eccentric hypertrophy enlarges the volume of cardiac chambers rather than increasing wall thickness and is seen in response to volume overload, such as in the case of valvular regurgitation. Both types of hypertrophies are mediated by some form of cell damage, which activates inflammatory signaling pathways, stimulating structural changes in the heart that may later lead to systolic and diastolic dysfunction [2]. Several signaling mechanisms are involved in the process of cardiomyocyte hypertrophy, including the upregulation of cytokines, hormones in the renin–angiotensin–aldosterone system (RAAS), fibroblast growth factors (FGFs), and other inflammatory signals [1]. Amongst these activated signaling pathways is the complement cascade.

Complement proteins are found in blood plasma and on cell surfaces, and they play a significant role in the initiation of the innate immune response during phagocytosis and opsonization [3,4]. Three activation pathways comprise the complement cascade, denoted as classical, lectin, and alternative [5]. The classical pathway of the complement system is primarily activated via the binding of antigen–antibody complexes, specifically those involving IgM and IgG antibodies, to the C1q protein. This interaction initiates a cascade that leads to the formation of the membrane attack complex, which can lyse target cells and promote their clearance. The binding of C1q activates C1r and C1s, a pair of proteases within the C1 complex. Activated C1s further cleaves C4 and C2, leading to the formation of the C3 convertase C4b2a. The C3 convertase cleaves C3 into C3a and C3b. The C3b then binds to the convertase to form a C5 convertase (C4b2a3b), which cleaves C5. This initiates the final steps of the pathway, ultimately leading to the assembly of the membrane attack complex (MAC). The MAC forms a pore in the target cell’s membrane, triggering cell lysis and death. 

The lectin pathway is initiated by mannose-binding lectin (MBL) and its associated serine proteases (MASPs). Crucially, MASP-1 activates MASP-2, which then cleaves the C4 and C2 complement components to form a C3 convertase complex (C4b2a). The initiation of the alternative complement pathway occurs due to the spontaneous hydrolysis of C3, forming C3(H2O). This molecule then binds to factor B, and factor D cleaves to form the C3 convertase (C3(H2O)Bb), triggering a cascade of events that eliminate pathogens and promote inflammation. The C3 convertase cleaves additional C3 into C3b and C3a. Newly formed C3b can attach to pathogen surfaces, where it also binds factor B and is cleaved by factor D to form another C3 convertase (C3bBb). This creates a potent positive feedback loop that amplifies the complement response. The C3bBb complex then binds another molecule of C3b and properdin to form the C5 convertase C3bBbP. The C5 convertase cleaves C5 into C5a and C5b. C5b then initiates the formation of the MAC by recruiting C6, C7, C8, and C9 [4]. C4b-binding protein (C4BP), composed of C4bpA and C4bpB subunits, inhibits the complement system’s classical and lectin pathways to limit inflammation and damage to host tissue. By binding to activated component C4b, C4BP prevents the formation of key enzyme complexes, reducing the production of C3a and C5a anaphylatoxins and blocking the assembly of the MAC [5].

Traditional activation of the classical pathway by immunoglobulin binding is involved in cardiomyopathy in helper T cells (Th1)-like autoimmune conditions. Autoantibodies, specifically IgG2/3, produced against the self-antigens of cardiac proteins such as myosin and beta-1 adrenergic receptor, have been reported in idiopathic cardiomyopathy and dilated cardiomyopathy patients [6,7]. The sera were also positive for several pathogenic antigens, including streptococcus cytomegalovirus antigens, implicating these subclinical infections as pathogenic triggers that contribute to the severity of cardiomyopathy [6]. Mechanistically, the Th1 response enhances IL-12-mediated humoral immunity with upregulation of IgG subclasses, specifically IgG2a, to result in classical complement pathway activation [8]. In patients with end-stage heart failure, total IgG levels in myocardial lysates, along with the infiltration of T cells, B cells, and macrophages into the heart muscle, correlated with increased complement activation marker C3c deposition. This was observed in both ischemic heart disease and dilated cardiomyopathy [9].

Pre-clinical models have also demonstrated that hypertension and increased angiotensin II levels correspond with an increase in complement activation, suggesting that the complement system plays a role in cardiac remodeling in response to these pathologic conditions [10]. Pathological damage to the heart can occur through various means. In myocardial infarctions, cardiomyocytes are deprived of nutrients and oxygen, causing ischemic damage. In volume or pressure-overloaded states, hemodynamic instability can create mechanical stress that damages the endothelial lining of the heart. Upon cell damage, damage-associated molecular patterns (DAMPs) are released [11]. The presence of DAMPs, or molecules released by dying cells, activates the complement system and subsequent inflammatory response [12]. While the classical pathway is most known for its activation by antibody–antigen complexes, other triggers exist, including certain molecules released from damaged or dying host cells. Instead of binding to an antibody, DAMPs like the C-reactive protein, heat shock proteins, proteins released from necrotic and apoptotic cells, and viral antigens such as Epstein–Barr virus, bind directly to the C1 component of the classical complement pathway and specifically to the C1q molecule. C1q is a pattern-recognition receptor that can bind directly to specific non-antibody molecules. When C1q binds to these DAMPs, it prompts a conformational change that activates the associated C1r and C1s proteases, thus initiating the classical complement cascade [13,14]. Endothelial cells themselves have receptors for complement anaphylatoxins, such as C3a receptor and C5a receptors 1 and 2. When activated, these receptors further mediate downstream inflammatory signaling and vascular remodeling in the setting of pulmonary hypertension [15]. The underlying basis of cell damage as a trigger of complement activation, inflammation, and eventual vascular changes can similarly apply to the cardiac hypertrophy model to characterize the role the complement system plays in pathological cardiac remodeling. Such damage to the heart can be initiated by autoimmune, infectious, and non-infectious etiologies, as will be further discussed in this review.

## 2. The Role of Complement Factors in Cardiac Inflammation and Hypertrophy

### 2.1. Classical Pathway

As previously described, the classical pathway of the complement system involves the binding of antigen–antibody complexes to C1q [3]. This interaction initiates a cascade of downstream effects, including the production of opsonin C3b and inflammation-mediating anaphylatoxins C3a and C5a [5]. A 2020 study suggests that C1q regulates cardiac hypertrophy in the context of progranulin (PGRN) deficiency [16]. PGRN is a circulating glycoprotein with anti-inflammatory effects that has previously been studied in the context of neurodegenerative diseases, as PGRN levels decrease with age [17]. Less well defined is the relationship between PGRN, aging, and the complement system. A study published in 2020 demonstrated that PGRN KO mice, or “aged” mice, were shown to have increased levels of C1q as compared to the WT group [16]. Upregulated C1q then activated cardiomyocyte β-catenin downstream, and the mice showed a significant increase in levels of fibrotic signals (collagen type I alpha 1 chain and collagen type III alpha 1 chain) as well as marked cardiac hypertrophy. Cardiac hypertrophy was quantified by collagen deposition in the interstitial fibrotic area, visualized by Masson’s trichrome staining, and an increase in cardiomyocyte size, quantified via an anti-α-actinin antibody stain. Markers of cardiac stress, atrial natriuretic peptide (ANP) and brain natriuretic peptide (BNP), were also significantly increased in the PGRN knockout (PGRN KO) mice as measured by mRNA expression in real-time PCR [16]. These studies collectively suggest that PGRN itself has a cardio-protective role. However, low levels of PGRN can result in high levels of C1q, therefore contributing to an inflammatory response that eventually leads to cardiac hypertrophy. Specifically, C1q activates β-catenin to subsequently upregulate expression of c-myc, c-fos, and transcription factor 4, which are all involved in cell proliferation, differentiation, and growth processes [16]. Since PGRN naturally decreases with age, this pathway characterizes the pathogenesis of C1q-driven cardiac hypertrophy related to aging. This demonstrates one avenue by which activation of the classical pathway of complement results in cardiac consequences. These PGRN KO mice hearts also exhibited increased C1q and β-catenin protein expression levels, and administration of C1q inhibitor to old KO mice reduced cardiac hypertrophy and dysfunction [16]. Furthermore, emerging pre-clinical research suggests that PGRN also plays a role in maintaining hemodynamic stability [18]. Although further investigation should focus on characterizing the specific pathways through which PGRN affects vascular tone and blood pressure, data from PGRN KO mice suggest that it exerts an anti-contractile function in the vasculature as well as induces synthesis of the potent vasodilator nitric oxide [18]. Another study performed on PGRN-ApoE double-knockout mice found that the lack of PGRN in the context of a high-fat diet contributed to more severe atherosclerosis compared to that of mice with the ApoE knockout alone. In the context of known strong PGRN expression in the foam cells of human atherosclerotic plaques, this further suggests a relationship between PGRN expression and cardiovascular health [19].

### 2.2. Lectin Pathway

The lectin pathway initiates via the interaction between mannose-binding lectin (MBL) and pathogen-associated molecular patterns (PAMPs) [20]. Additionally, MBL-associated serine proteases (MASPs) are involved in the activation and amplification of this pathway. MBL-PAMP binding triggers autoactivation of MASP-1, which subsequently activates MASP-2 to cleave C2 and C4 and contribute to the formation of C3 convertase C4bC2a [20]. A 2024 study suggests that the lectin pathway can interact with the coagulation cascade by MASP-1-induced clot formation in normal citrated plasma, influence on clot structure, and prolongation of clot lysis. Researchers demonstrated that MASP-1 directly activates prothrombin, factor VIII, and thrombin-activatable fibrinolysis inhibitor. It also induces fibrin formation in a thrombin-dependent process [21]. In the context of cardiovascular disease, and especially myocardial infarction, the interplay between the complement system and coagulation cascade may present a potential therapeutic target.

A comparative study focused on levels of MBL and MASPs in patients with cardiovascular and cerebrovascular diseases observed that patients with a recent myocardial infarction had higher serum MASP-1 levels than healthy controls, coronary artery disease (CAD) patients, and stroke patients. Additionally, MASP-2 levels were higher in the group of patients with CAD than in the stroke, MI, and healthy control subpopulations [22]. These findings suggest that the lectin pathway may contribute to or be activated in the context of cardiovascular disease. Another study on MASP-2 double-knockout mice questioned the role of MASP-2 and the lectin pathway in the pathogenesis of post-ischemic reperfusion injury of the heart. The MASP-2-deficient mouse hearts experienced significantly less tissue damage compared to the WT hearts after an induced period of transient ischemia and reperfusion [23]. MBL-associated protein MAp44 is highly expressed in cardiac tissue, and previous research on murine models shows that it may act as a cardioprotective factor by competing with MASP-2. MAp44 is a phylogenetically conserved protein with eight exons coding shared domains with MASP-1 and MASP-3, and the ninth exon is unique to the structure of MAp44. Interestingly, plasma MAp44 levels remain unaltered in patients with acute myocardial infarction, with elevated MASP-1 levels suggesting a poor protective response by MAp44 [24]. Conversely, patients with coronary artery disease showed a positive correlation between plasma MASP-2 and MAp44 levels, indicating a compensatory mechanism played by MAp44 to inhibit myocardial damage. Furthermore, myocardial infarctions lead to tissue death and subsequent adverse cardiac remodeling, especially of the left ventricle, which may lead to heart failure [25]. As it appears that the lectin pathway is a major contributor to myocardial damage and fibrosis after ischemic events, additional research into ways this pathway may be inactivated or protected against may prevent adverse outcomes after myocardial infarctions and similar insults.

### 2.3. Converging Point: C3

Complement component 3, or C3, is the point at which all pathways of complement activation converge, and its cleavage results in the production of anaphylatoxins C3a and ultimately C5a [3]. Elevated levels of C3 and complement dysregulation contribute to various pathologies, including those impacting the cardiovascular system. For instance, a study of 7820 patients without hypertension linked elevated serum C3 levels to the prevalence of prehypertension [26]. A different study published in 2019 on 80,517 individuals without venous thromboembolism found that increasing concentrations of plasma C3 were associated with a proportional increased risk of venous thromboembolism, with similar results in patients with deep venous thrombosis and pulmonary embolism. The researchers suggest that an interaction between the complement system and the coagulation cascade may have led to this finding [27].

Looking more specifically at the heart, one study examined the effects of injecting purified human C3a into guinea pig hearts. These intracoronary boluses led to tachycardia, impairment of the electrical conduction system, systolic failure of the left ventricle, coronary artery vasoconstriction, and release of the vasoconstrictor histamine. Given that common disease states can easily approximate the C3 concentrations administered, the data indicate a role for the generation of anaphylatoxins in the pathogenesis of cardiac dysfunction in these illnesses [28].

Moreover, plasma C3a levels showed positive correlations in the progression of clinical events to congestive heart failure (CHF) and mortality in a 14-month follow-up study post-CHF in 182 patients. Plasma C3a levels were more significantly associated with these events than the soluble terminal membrane attack complex (SC5b9), a significant predictor of heart failure. Also, C3a levels positively correlated with peripheral edema and systemic inflammatory mediators, including IL6, prealbumin, and CRP, and negatively correlated with HDL-cholesterol [29]. A different study on 302 human patients with coronary artery disease revealed a positive correlation between C3aR and C5aR1 platelet expression and platelet activation, thus indicating that C3a and C5a contribute to the pathogenesis of coronary artery disease. Patients with acute myocardial infarctions also showed elevated C5aR expression on platelets but demonstrated similar C3aR levels as healthy controls [30]. A 2022 study examined the role of complement in right heart failure, specifically, as it significantly contributes to the risk of mortality in patients with existing left ventricular failure. Transcriptome analysis comparing gene expression patterns between the ventricles and subsequent ingenuity pathway analysis showed high expression of complement genes *Cfd*, *C3*, and *C3ar1* in the right ventricles of mice. This finding also extends to human cardiac tissue, where CFD, C3, and C3aR1 are similarly upregulated. To investigate changes in complement in pathological conditions, researchers induced pulmonary artery constriction (PAC) in mice to cause right ventricular failure. Pulmonary artery constriction resulted in increased *Cfd* and *C3ar1* expression and the presence of C3 in the right ventricle (RV), which suggests a possible contribution of complement activation to the development of RV failure [31].

One mechanism by which C3a causes cardiac dysfunction, as modeled in guinea pigs, involves C3a/C3aR signaling-mediated histamine and leukotriene release, thereby causing tachycardia, impaired contractility, and vasoconstriction. Antagonistic treatments with antihistamine and anti-leukotriene using histamine H_2_ receptor antagonist cimetidine and FPL-55712, respectively, rescued guinea pig hearts from the effects of intracoronary C3a administration. Moreover, carboxypeptidase B-mediated lysis of the C-terminal arginine plausibly reduced the affinity of the C3a-desArg binding to its cognate receptor C3aR, resulting in attenuation of cardiotoxic effects of C3a [28]. In summary, the overexpression of C3 and its anaphylatoxin products may adversely affect the cardiovascular system.

### 2.4. Alternative Pathway

The alternative pathway is characterized by activation via spontaneous C3 hydrolysis and is unique in that it amplifies activation of the other two pathways [32]. Dysregulation of the alternative pathway has been implicated in a variety of cardiovascular diseases. For instance, patients with carotid atherosclerosis exhibit increased levels of properdin compared to healthy controls. And yet lower levels of properdin, a positive regulator of the alternative pathway, are linked to a higher rate of cardiovascular mortality in this same population. Such findings seem contradictory, as other research has pointed to an association between excessive complement activation via all three pathways [32,33]. This only additionally emphasizes that the complement system has a complex role that requires further study.

Other studies have examined the relationship between the alternative pathway and heart failure. One study compared 343 patients with heart failure to healthy controls, and researchers found increased C3bBbP, the C3 convertase associated with the alternative pathway, in the plasma of those with heart failure [34]. A different study yielded similar findings, going further to correlate increased serum levels of complement factor D with increased heart failure severity. Interestingly, it was found that these patients with heart failure also had low levels of properdin [35]. Again, this demonstrates that the alternative pathway has an intricate relationship with cardiovascular disease pathogenesis.

### 2.5. Membrane Attack Complex

The membrane attack complex (MAC) is composed of complement proteins C5b through C9 [5]. In the setting of infection, the MAC assembles on the target pathogen membrane and creates pores, causing cell lysis and death [36,37]. Human cells have protective regulatory proteins that prevent MAC formation and subsequent lysis, but pathogens without such proteins are vulnerable to the activities of the MAC [38]. Additionally, the MAC triggers intracellular signaling pathways that increase levels of proinflammatory factors such as cytokines, cellular adhesion molecules, and tumor necrosis factor-α (TNF-α) [36,38,39]. In terms of the vasculature specifically, MAC proteins C5b-C9 have been found to induce proinflammatory mediator interleukin-6 (IL-6) in vascular smooth muscle cells via nuclear factor kappa-light-chain-enhancer of activated B cells (NF-κB) and activator protein-1 (AP-1) upregulation, which may contribute to vascular damage [40]. Thus, the MAC exhibits inflammatory potential in the pathogenesis of cardiovascular diseases.

Earlier research found that 28 patients with dilated cardiomyopathy, a form of eccentric hypertrophy, also had elevated levels of C5b-C9 in their myocardial tissues upon biopsy. The presence of C5b-C9 correlates with deposition of immunoglobulins and expression of TNF-α in myocardium, and TNF-α activity is heavily implicated in the progression of cardiac damage and disease, as it precipitates a negative inotropic response in myocardium and even encourages apoptosis of cardiac myocytes [41]. Another study on patients with end-stage heart failure yielded similar findings. Researchers performed biopsies before and after left ventricular assist device (LVAD) transplantation and compared results to normal, healthy cardiac tissue. The heart failure tissue contained MAC deposition in the interventricular septa, the ventricles, and the apexes, while the healthy hearts exhibited no traceable amount of MAC deposition. After patients received an LVAD, the amount of MAC deposition decreased, as visualized by immunohistochemistry and Western blotting [39]. The LVAD is a machine indicated for patients with severe systolic dysfunction and essentially acts in place of the left ventricle to funnel blood from the heart to the rest of the body. While the heart still contracts and beats, it is no longer the primary driver of systemic blood flow [42]. LVADs, therefore, reduce the mechanical burden of the heart. The results of the study suggest that myocardial strain is associated with MAC deposition and that alleviation of this strain via implantation of an LVAD decreases complement activation and deposition. Given the MAC contribution to cellular damage, lysis, apoptosis, and upregulation of factors like leukotrienes and TNF-α that are known to cause tissue remodeling, terminal complement factors represent an intriguing therapeutic target for those with heart failure [39]. Selective prevention of MAC deposition and subsequent upregulation of inflammatory markers may significantly avert further cardiac damage in this population.

## 3. Regulators of Complement Activation

### 3.1. Soluble Regulators

Regulators of the complement system include several cofactors, proteinases, and cell membrane receptors that can cleave or otherwise inactivate constituents of the complement cascade [43]. Complement factor I (CFI), for instance, is a freely soluble serine proteinase capable of cleaving C3b and C4b to render them inactive and prevent the creation of C3 convertase. Another regulatory protein of note is complement factor H (CFH), which works in tandem with CFI as a cofactor that preferentially binds to C3b complexed to the host to downregulate complement activation and protect the host from inflammatory damage [3]. Conversely, complement factor B (CFB) preferentially binds to C3b, which binds to foreign cells such as bacteria or fungi to trigger phagocytosis and amplify the inflammatory response [3]. This CFH-CFB relationship allows the body to create either a positive or negative feedback response that ultimately works to protect the host while killing pathogens.

Another regulator of complement, factor D (CFD), serves as the rate-limiting step in the formation of alternative pathway C3 convertase. [36] Deficiencies in CFD are linked to increased risk of recurrent infection, as the immune system is no longer properly primed to respond to foreign antigens [44]. Complement factor P, also known as properdin, has a similar function in supporting complement activation by stabilizing the newly formed C3 convertase in the alternative pathway [43]. In terms of cardiac-specific consequences, patients with heart failure have been found to exhibit increased serum levels of factor D. As factor D is integral to the downstream activation of the alternative pathway, the complement system is implicated in the progression of cardiac failure. An excess of such positive regulatory proteins may be harmful [35].

### 3.2. Membrane-Bound Regulators

An example of a cell membrane receptor involved in regulation is complement receptor 1 (CR1/CD35), which is found on phagocytes, B-cells, antigen-presenting cells (APC), and other blood and immune cells [43]. CR1 on erythrocytes clears immune complexes from the body, preventing an excessive inflammatory response. On the other hand, complement receptor 2 (CR2, also known as CD21) is found on B lymphocytes and promotes antigen processing and B cell activation, leading to more antibody production via interaction with an antigen-C3d complex [45]. Complement receptor 3 (CR3) mainly has a role in phagocytosis, especially of pathogens tagged for opsonization [43].

**Figure 2:**
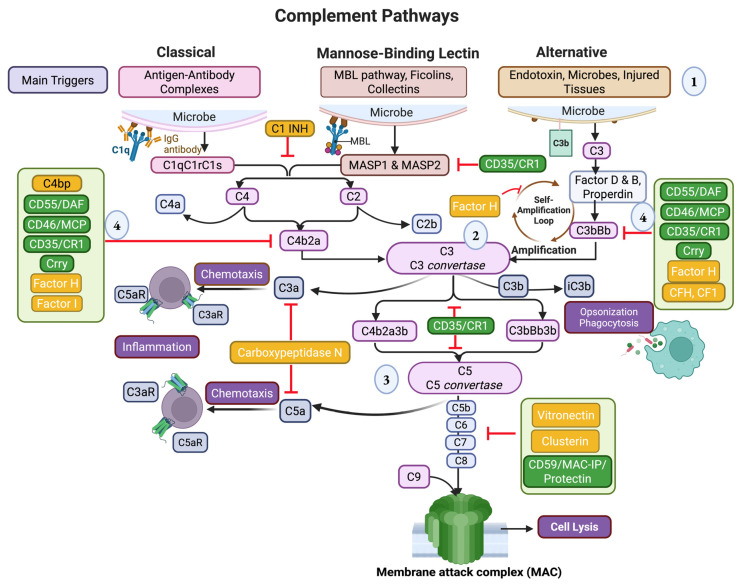
The three complement cascades. The three complement cascades are the classical pathway, the lectin pathway, and the alternative pathway (1). These pathways comprise the complement system, a part of innate immunity that is activated to combat pathogens. They all converge at a point where a key enzyme called C3 convertase forms (2), leading to a cascade of events that result in pathogen destruction. Subsequent cleavage of complement components C3 and C5 leads to the assembly of a membrane attack complex (MAC) that creates pores in the pathogen’s membrane, triggering cell lysis (3). A variety of membrane-bound inhibitory molecules, including CD55, CD46, CD59, Crry, and soluble factors C4bp, Factor H, Factor I, prevent excessive activation and damage to host cells (4). These inhibitors target different stages of the cascade from the initial recognition of pathogens to the alternative complement pathway and the formation of MAC.

Another regulatory protein within the cell membrane is the membrane cofactor protein (MCP/CD46). Its role is to aid CFI as a cofactor in preventing the activation of C3 convertase [46]. Perhaps the most well-known membrane-bound regulators of the complement system are the decay-accelerating factor (DAF/CD55), which binds C3b and C4b to prevent synthesis of C3 convertase, and membrane inhibitor of reactive lysis (MIRL/CD59), which binds to C9 and prevents the formation of the MAC [38]. More importantly, these regulatory proteins control the complement system and prevent it from damaging self-cells. CD59 is the only membrane-bound inhibitor of MAC formation in human cells [47]. It plays a crucial role in preventing MAC-mediated lysis by binding to the developing MAC and disrupting its assembly. Thus, the presence of these protective regulatory proteins is essential for maintaining cell integrity and preventing harmful bystander damage from the complement system’s defensive actions (Figure 2). Deficiencies in DAF and MIRL on red blood cells are a prime example of why proper regulation of the complement cascade is essential, as these deficiencies lead to a condition known as paroxysmal nocturnal hemoglobinuria (PNH). In PNH, inadequate levels of DAF and MIRL mean that C3 convertases and the MAC are constitutively expressed, leading to inappropriate complement activation and excessive lysis of erythrocytes, especially at night when the body is in a more acidotic state [38]. Patients with PNH thus experience red or dark brown urine during the night or first thing in the morning due to this intravascular hemolysis. This hematologic disorder demonstrates how a lack of negative regulatory proteins can allow the complement system to attack normal, healthy host cells, which can lead to further complications such as anemia.

Regulators of the complement cascade collectively act in the host’s best interest by ensuring that complement activation occurs in the presence of a foreign invader. It also modulates and de-escalates the inflammatory response to protect the host from damaging itself. Using this reasoning, cardiac inflammation and hypertrophy can be caused by either overactivation of the complement cascade or a loss of negative feedback regulation [11]. Figure 2 illustrates the three complement pathways and the regulators of complement activation involved in opsonization and phagocytosis of damaged host cells and pathogens. 

## 4. Genetic Variations in Complement Genes and Cardiac Consequences

### 4.1. Complement Component 2

The complement system comprises over 40 proteins, both freely circulating and membrane-bound [48]. Genetic polymorphisms in a multitude of these proteins have been associated with cardiac consequences. For instance, a variant in the C2 gene caused by a deletion of 28 base pairs leads to C2 deficiency (C2D) type I [49]. This genetic variation is the primary cause of C2D, accounting for approximately 90% or more of C2D cases in humans [50]. A study over the span of 25 years on 40 people in Sweden with C2D found that these patients exhibited an expected increased susceptibility to infections but also a higher risk for acute myocardial infarctions compared to the general Swedish population, although the coronary risk profile was not controlled for. Researchers postulate that there is a relationship between C2D and the development of atherosclerosis, although the exact mechanism by which this occurs remains unclear and warrants further study [50].

### 4.2. Complement Factor H

Complement factor H (CFH) genetic variants have been associated with several disease processes, most notably atypical hemolytic uremic syndrome (aHUS); approximately 48 mutations in the CFH gene have been found to cause aHUS [51]. Typical hemolytic uremic syndrome is most commonly due to infection with *Escherichia coli*, which releases a toxin that ultimately causes intravascular hemolysis and kidney failure [52]. However, emerging evidence suggests that the CFH gene may be responsible for more than just aHUS. For instance, five variants in the CFH gene (L3V, R127H, R166Q, C1077S, and N1176K) were identified in women with preeclampsia but not among controls. These results suggest that the CFH gene may influence the cardiovascular system [53].

The CFH Tyr402His (CFH Y402H) functional polymorphism in the seventh consensus repeat region of the gene results in decreased binding between its product and C-reactive protein (CRP) [54]. In normal circumstances, the interaction between CFH and CRP helps regulate complement activation by facilitating the elimination of cellular debris to reduce inflammation. Thus, the CFH Y402H polymorphism leads to an unintended and poorly controlled inflammatory response [55,56]. While this polymorphism has previously been linked to macular degeneration, a study (embedded within the Rotterdam Study) of 5520 patients over the age of 55 found a relationship between CFH Y402H and myocardial infarctions as well [57]. Contrarily, a different cohort study of people with familial hypercholesterolemia suggested that this polymorphism may be protective and decrease the risk of cardiovascular disease in this specific population [58]. The role of CFH polymorphisms in cardiovascular disease is therefore not entirely clear, though a direct relationship between the two seems to exist. Further research should explore the effects of CFH polymorphisms on the cardiovascular system while accounting for possible confounding variables and the interplay between the complement system and other immune processes.

### 4.3. Collectin-11 and MBL-Associated Serine Proteases (MASPs)

Collectin-11 (COLEC11), a pattern-recognition molecule, is an activator of the complement system via the lectin pathway upon binding to MASP proteins [59]. It also plays a role in embryonic development [60]. Additionally, COLEC11 can contribute to the growth and proliferation of cancer cells [61].

Collectin-10 (COLEC10), COLEC11, and MASP1/3 polymorphisms have been identified in Mingarelli, Malpeuch, Michels, and Carnevale (3MC) syndrome [62,63]. 3MC syndrome is a rare autosomal recessive disorder that results in widespread embryonic defects, including cleft palate, hearing loss, and umbilical hernias [62]. Although rare, these defects can include cardiac anomalies [63]. COLEC11 and MASP1/3 encode proteins expressed in the lectin pathway, and specifically, the protein CL-K1 is of interest in our discussion. Research on zebrafish embryos indicates CL-K1 is responsible for neural crest cell migration, acting as a chemo-attractant. Losses of the COLEC11 gene or the MASP1 gene, whether independently or in combination, led to defects in zebrafish embryos [63]. Therefore, complement genes and their protein products play a part in embryogenesis and can affect the development of craniofacial features and organ development, such as that of the heart.

However, collectin and MASP can have other cardiac-related effects besides those in the embryonic phase. A cohort study on 251 patients with Chagas disease in Brazil found that genetic variations in COLEC11 and MASP2 contributed to the pathogenesis of Chagas disease, which is characterized by dilated cardiomyopathy [61]. The interaction between the COLEC11 (rs7567833G>A) and MASP2 (g.1961795C>A, p. D371Y) polymorphisms is thought to behave synergistically in reaction to the parasite that causes Chagas disease, *Trypanosoma cruzi*, and this enhanced complement response subsequently contributes to cardiomyopathy [61].

### 4.4. C5a Receptor 1

Though C5aR1 is often discussed in terms of broad inflammatory responses, its expression may have specific beneficial cardiovascular effects. In preclinical studies on animals capable of cardiac regeneration after injury, such as zebrafish, complement receptor C5aR1 was found to be upregulated in cardiomyocytes and played a role in cardiomyocyte proliferation. On the other hand, C5aR1 KO animals displayed greater fibrosis and scarring upon regeneration compared to WT mice in response to injury [64]. Although human hearts lack this regenerative capacity, the findings of this study encourage more research into how the complement system influences cardiomyocyte response to injury. Additionally, C5aR1 has other effects on the cardiovascular system. A study published in 2015 showed a significant association between a C5aR1 polymorphism (rs10853784) and coronary artery disease in a Han population from Xinjiang, China [65]. Polymorphisms in C5a-like receptor 2 (C5L2) amongst the same population exhibited a similar correlation with disease risk [66]. Altogether, these studies suggest that C5aR1 expression exerts effects on cardiovascular responses to damage and risk factors that contribute to cardiovascular disease. The interaction between C5a and C5aR1 particularly activates neutrophils and monocytes; in fact, C5aR1 signaling on monocytes/macrophages directly contributes to subsequent angiotensin II-induced pathological cardiac remodeling. Broadly, C5aR1 expression and binding on atherosclerotic lesions cause local inflammatory responses that exert destructive effects on blood vessels, contributing to coronary artery disease and the possible development of future myocardial infarction [65].

### 4.5. Genetic Dysregulation Affecting the Alternative Pathway in Cardiovascular Diseases

Genetic dysregulation of the complement system contributes to cardiomyopathies primarily by altering the activation and regulation of the alternative pathway. This leads to excessive complement activation, chronic inflammation, and direct myocardial injury. Genetic variants encoding complement components or regulators, such as C3, CFD, and CFH, have been shown to modulate the activity of the alternative pathway. C3 deficiency is linked to increased post-infarction myocardial dysfunction, scar size, and left ventricular dilation. While C3 deficiency does not cause heart disease itself, its absence can worsen outcomes in those who develop cardiac issues, suggesting C3 plays a role in cardiac repair and inflammatory response after injury [67]. Deficiency of CFD with two mutations at T638>G (Val213>Gly) and a T640>C (Cys214>Arg) mutation in the genomic DNA from the patient, both in homozygous form, abolishes the catalytic activity of the CFD in forming C3 tick-over convertase (C3bBb) and therefore impairs alternative complement pathway activation. Reduced activation increased susceptibility to invasive meningococcal infections in patients, leading to acute meningococcal myocarditis [68,69]. While a deficiency of CFD is rare, studies show that increased circulating levels of CFD lead to cardiovascular issues such as vascular damage, inflammation, and endothelial dysfunction that can contribute to cardiomyopathy [31,35]. CFH mutations as described above are substantially studied in the context of age-related macular degeneration and other autoimmune and thrombotic diseases [70]. However, a mutant CFH phenotype is a rare cause of acute systolic heart failure in patients with aHUS. The 24-year-old young patient presented with significantly lower C3 and C4 levels along with thrombocytopenia, and skin biopsy showed thrombotic microangiopathy [71]. Combinations of these variants, sometimes termed “complotypes”, can synergistically enhance only result in a modest effect; their combined presence can tip the scales of complement regulation toward sustained activation, which has been implicated in complement-mediated diseases and may similarly predispose individuals to cardiomyopathy [72,73]. Interestingly, the C3-Cfd-C3aR axis (a pathway involving C3) plays a crucial role in right ventricular failure, with C3-knockout mice showing attenuated dysfunction and fibrosis [31]. 

In dilated cardiomyopathy, chronic complement activation, often triggered by autoimmune or post-viral injury, leads to myocardial deposition of the MAC and subsequent increased TNF expression, local inflammation, and adverse remodeling. These effects contribute to progressive myocyte apoptosis and chamber dilation [41]. Likewise, aberrant C5aR1 pathway activation can exacerbate myocardial injury in arrhythmogenic cardiomyopathies, as C5a–C5aR1 interactions recruit neutrophils and monocytes, promote tissue fibrosis, and enhance electrical instability. Notably, preclinical studies show that genetic or pharmacologic inhibition of C5aR1 reduces arrhythmogenesis [74,75].

Moreover, patients with heart failure have shown increased levels of alternative pathway activation products like C3bBbP and CFD, correlating with disease severity and worse outcomes [31,35]. Overall, these findings support the notion that genetic variants which amplify alternative pathway activation or impair its regulation drive chronic inflammation, fibrosis, and cardiomyocyte dysfunction, therefore contributing to the development and progression of cardiomyopathies.

## 5. Autoimmune Diseases, Complement Activation, and Cardiac Consequences

### 5.1. Systemic Lupus Erythematosus

Systemic Lupus Erythematosus (SLE) is a systemic autoimmune disease that targets connective tissues and exerts cardiac effects, including inflammation and damage of the heart, pericardium, and vasculature [76,77]. In fact, over half of patients with SLE experience cardiac manifestations [78]. Previous immunofluorescence studies found C3 deposition in the pericardium, suggesting complement system involvement in the pathogenesis of cardiac disease related to SLE [76]. Plasma levels of C4d have previously been found to serve as a strong biomarker of SLE disease activity, with utility in identifying patients with lupus nephritis. Levels of C4d indicate higher versus lower disease activity, and at higher levels correlate directly with the modified SLE Disease Activity Index [79]. At the same time, patients with SLE exhibit lower serum levels of C3 and C4 due to a higher rate of complement consumption in the context of complement dysregulation and alternative pathway activation [77,80]. This relationship is significant enough that decreased complement proteins comprise an immunologic criterion in multiple existing classification criteria for SLE. That said, a small subset of patients with SLE suffer from concurrent primary complement deficiency and may exhibit low complement levels at any stage of disease progression or clinical presentation [80]. The results of case–control and follow-up studies suggest a close relationship between leukocyte expression of the membrane-bound complement regulatory proteins CD46, DAF, CR1, and CD59 transcripts and disease activity in SLE. Specifically, individuals with SLE exhibited reduced CR1 expression [81]. CR1, responsible for binding and clearing immune complexes in circulation, is also expressed at lower levels on erythrocytes of those with SLE [82]. A second study examining the relationship between the presence of single-nucleotide polymorphisms (SNPs) in the CR1 gene and subsequent risk of MI identified 12 SNPs corresponding to significantly increased risk of both fatal and nonfatal MI [51]. As SLE results in immune complex deposition throughout the entire body, there is potential that such variations in complement genes can also correlate with cardiac manifestations of SLE [76].

A study published in 2022 discovered that pediatric patients with SLE who had a gene copy number of C4b ≥ 2 suffered a higher incidence of hypertension and pericarditis, although the exact mechanism behind this relationship has yet to be elucidated [83]. SLE also influences atherosclerosis pathogenesis. In a study of 214 women with SLE and no known cardiac complications, aortic stiffness positively correlated with higher serum C3 complement levels [84]. Thus, complement is implicated in cardiac inflammation and damage and contributes to the risk for chronic damage and hypertrophy of the heart. The expression of complement receptors on atherosclerotic lesions leads to an inflammatory response that causes vessel remodeling [65]. Therefore, high levels of complement expression might correspond to a greater rate of atherosclerosis when investigated further.

In an experimental study conducted in MRL/lpr mice (MRL/MpJ-Fas lpr/J) that develop SLE-like disease, Botto M et al. [85] demonstrated that deletion of C1q induced severe proliferative and crescentic glomerulonephritis, immune bodies, and high titers of autoantibodies. Moreover, they also showed significantly increased glomerular apoptotic bodies in mice lacking the glomerulonephritis phenotype, suggesting that inherited deficiency of C1q may contribute to clearance failure and thus increased deposition of apoptotic bodies, stimulating autoantibody response. Paradoxically, the intact C3 in these C1q-deficient mice could augment the alternative complement pathway, thereby exacerbating glomerulonephritis. Using the mouse strain, an independent study demonstrated the protective role of both inducible NOS2 and endothelial-specific NOS3 nitric oxide synthases in preventing aortic lesions. The mechanism underlying pathologic consequences in mice lacking either NOS was attributed to lipoprotein immune-complex deposition, increased sphingosine-1-phosphate (S1P), and decreased anti-inflammatory cytokine IL-10 [86]. Perhaps these two studies indirectly link proinflammatory pathways and reactive oxygen species generation and help extrapolate explanations behind lupus-associated augmentation of cardiac remodeling and hypertrophy.

### 5.2. Systemic Sclerosis

Systemic sclerosis (SSc) is another autoimmune disease and a subcategory of scleroderma that affects various body tissues and organs, including the heart. It causes fibrosis that ultimately impairs systolic and diastolic function [87,88]. Most scleroderma patients present with thickening or hardening of the skin. Still, pulmonary fibrosis, pulmonary arterial hypertension, and cardiac disease comprise the major disease-related causes of death in patients with SSc specifically [89]. Apart from the most common antinuclear antibody (ANA) present in 90% of scleroderma patients, the European League Against Rheumatism/American College of Rheumatology (ACR/EULAR) classification also identifies more SSc-specific autoantibodies like anti-centromere antibodies (ACAs), anti-topoisomerase I antibodies (ATAs), and anti-RNA-polymerase III antibodies (ARAs). Specific antibodies can correspond with different clinical features and disease courses. For example, patients with ACAs are more likely to present with limited skin involvement, while those with anti-Scl-70 are more likely to possess diffuse skin and organ involvement [90,91,92]. There is no agreed-upon definition of SSc cardiac sequelae, though known symptoms include arrhythmias, heart failure, and pericardial disease. Upon autopsy, approximately 80% of patients with SSc display cardiac manifestations of disease in the form of hypertrophy, myocardial contraction band necrosis, myocardial lesions, and pericardial fibrosis [88,93,94]. While patients infrequently present with cardiac symptoms, the complement system may play a role in adverse cardiac manifestations of SSc [88,95].

The complement system is hyperactive in SSc patients, and this relationship has been studied in the context of more common presentations of the disease, such as pulmonary fibrosis and scleroderma renal crisis [96,97]. A 2016 study found that patients with SSc and scleroderma renal crisis exhibited significantly lower serum levels of C3bBbP and higher serum levels of C4d than those without renal manifestations [97]. A different study on skin biopsies from SSc patients found significant C5b-9 deposition, again suggesting that complement dysregulation contributes to various clinical presentations of SSc. Additionally, membrane cofactor protein (MCP/CD46) appeared to experience downregulation in vascular endothelial cells of these SSc patients [98]. Conversely, other studies found no correlation between serum C3 and C4 levels and skin and lung fibrosis [96]. In a cross-sectional study published in 2024, which consisted of 430 adult participants with SSc, elevated plasma levels of C5 were positively associated with atherosclerosis and calcification of the coronary arteries [99]. Although the mechanism remains unclear, it stands to reason that complement system activation, which has been correlated with arterial thickening and calcification, could also hold responsibility for the same effects on cardiac valves themselves and contribute to eventual cardiac remodeling. Thus, more research must be conducted to determine the nature of complement system activation and mediation of inflammation and damage to the heart or other organs, as well as to identify possible therapeutic targets.

### 5.3. Rheumatoid Arthritis

Rheumatoid arthritis (RA) is a systemic autoimmune disease that classically impacts joints but has myriad extra-articular manifestations, including those within the cardiovascular and pulmonary systems [100]. The pathogenesis of RA involves a wide array of immune cells, such as B cells, T cells, macrophages, and plasma cells, but most relevant to this review is the presence of anti-citrullinated protein antibodies (ACPAs), of which anti–cyclic citrullinated peptides (anti-CCPs) are a subset [100,101]. The presence of elevated anti-CCPs in conjunction with IgM-rheumatoid factor (RF) autoantibodies is mildly predictive of RA [101]. Importantly, ACPAs can bind to Fc receptors on myeloid cells, causing activation of the classical and alternative pathways of complement [102,103,104]. The presence of IgM and IgA RFs enhances the capacity of ACPAs to activate the complement system [105].

Furthermore, complement factor C3 is strongly associated with the cardiometabolic disease pathogenesis during the progression of RA and spondylarthritis. Thus, it serves as a biomarker reflecting disease activity in these rheumatic disorders [106]. Inhibition of inflammation with anti-rheumatic therapy using methotrexate and TNF inhibitors significantly reduced the presence of soluble terminal complement complex (TCC) and improved patient outcomes [107]. This implicates the complement cascade in the development of rheumatoid arthritis. Conversely, steroids and non-steroidal anti-inflammatory drugs (NSAIDs), such as rofecoxib (which has been removed from the market), enhance the cardiovascular risks in RA patients [108,109].

In the cardiovascular system, RA increases the risk of ischemic events, arrhythmias, heart failure, and pericarditis [110,111]. Activation of complement and TCC, led by the production of C3, factor B, and factor D (also known as adipsin) [112], by the perivascular and visceral adipose tissues is associated with cardiovascular events. The breakdown product of C3, C3desArg (also known as acylation-stimulating protein; ASP), is produced in adipose tissues and functions as an autocrine mediator of fat storage in the differentiated adipocytes during RA pathogenesis [113]. Proinflammatory cytokines and adipokines such as adiponectin, which binds to C1q, also activate the classical pathway and drive the pathogenesis of acute myocardial infarction [114,115]. Secondary amyloidosis due to RA can also contribute to cardiac hypertrophy [116]. RA is additionally associated with pericardial effusion as well as valvular dysfunction due to insufficiencies, thickening, calcification, and stenosis [109,117]. These studies suggest that RA patients have common risk factors associated with cardiovascular diseases, including hypertension, hyperlipidemia, and insulin resistance [118]. What requires further investigation is how complement activation may promote the development of cardiac disease in RA patients. Using single-cell transcriptome analysis, in wild-type C57B6/J mice with collagen-induced arthritis (CIA) and C5a induction for 30 days (WT-CIA+C5a), we identified pathways associated with dilated and hypertrophic cardiomyopathy. This was further supported by evidence of complement dysregulation. [119]. Analysis of cardiac cell populations in arthritis-bearing mice revealed a significant reduction in cardiomyocytes and a corresponding increase in endothelial cells and fibroblasts when contrasted with WT-PBS controls. While gene ontology analysis revealed increased protein synthesis, binding, and extracellular matrix production in these mouse hearts, KEGG pathway analysis mapped these genes to dilated cardiomyopathy and cardiac remodeling. More importantly, the WT-CIA+C5a mouse hearts showed significant upregulation of the glutamyl-prolyl-tRNA synthetase *Eprs1*, which functions in proline-rich collagen synthesis. This finding was accompanied by decreased mitochondrial cytochrome c oxidases (*mt-Co1*, *Co2*, *Co3*), most likely due to cardiomyocyte dysfunction and fibrosis [119]. Pathological eccentric hypertrophy, often caused by volume overload, is marked by cardiac chamber dilation and myocardial fibrosis. Since collagen and other ECM proteins are rich in proline, the upregulation of Eprs1 enhances their synthesis. Research shows that Eprs1 is upregulated at both the mRNA and protein levels in failing human hearts and in mouse hearts experiencing pathological cardiac remodeling [120,121].

### 5.4. Anti-Phospholipid Syndrome

Anti-phospholipid syndrome (APS) is an autoimmune disorder characterized by an array of systemic pathologies such as recurrent venous-arterial thrombosis, pregnancy loss, and thrombocytopenia due to the presence of anti-phospholipid antibodies (aPL). The aPL are generated not just against the anionic phospholipids, such as anti-cardiolipin and lupus anticoagulant (LA), but also circulating protein beta-2-glycoprotein-I (β2GPI), which interacts with the anionic phospholipids on the plasma membrane [122]. APS may present as a disorder secondary to SLE but can also be a primary disorder of its own. The presence of LA, as the key component of APS, is associated with miscarriages, thrombosis, and rare bleeding conditions, including brain hemorrhage, gastrointestinal bleeding, and diffuse muscular hemorrhage [122,123]. Though most APS effects occur in the vasculature, it can also impact the heart. There is an association between primary APS and cardiac valve lesions, thickening, and non-bacterial vegetations (known as Libman-Sacks endocarditis) [124].

**Figure 3:**
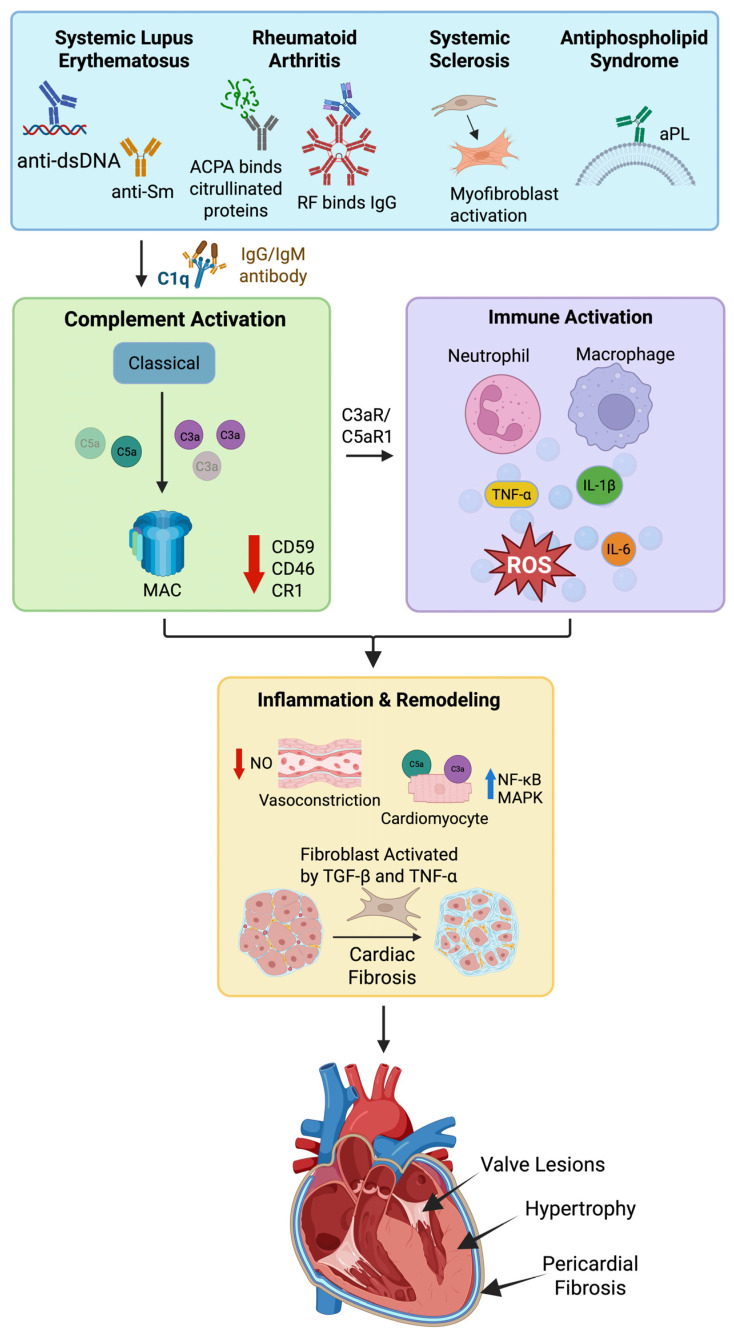
Role of complement in autoimmune disease-associated cardiac hypertrophy. Autoantibodies involved in autoimmune conditions that induce pericarditis include anti-dsDNA in anti-Smith antibodies in SLE. Anti-citrullinated protein antibodies (ACPA) and rheumatoid factor (RF) in rheumatoid arthritis are also associated with increased risk of chronic cardiovascular events and acute pericarditis. In the systemic sclerosis condition, antinuclear antibodies, anti-centromere antibodies (ACA), anti-topoisomerase I antibodies (ATA), and anti-RNA-polymerase III antibodies (ARA) contribute to the progression of acute pericarditis and pericardial effusion. Anti-phospholipid syndrome involves anti-phospholipid antibodies that target the phospholipid bilayer (Panel 1). These conditions induce classical complement cascade, increased alternative pathway activation, and generation of C3a and C5a anaphylatoxins with concomitant reduction in the complement regulators such as CD59, CD46, and CR1 (Panel 2). The anaphylatoxin receptor-mediated proinflammatory consequences lead to leukocyte infiltration and subsequent myofibroblast activation, resulting in cardiac tissue remodeling and hypertrophy (Panel 3).

Other rare cardiac consequences of APS include intracardiac thrombi, systolic and diastolic dysfunction, and myocardial infarctions [125]. The complement system may contribute to this interplay between APS and cardiac disease. In Libman-Sacks endocarditis associated with APS, vegetations on heart valves partly consist of immune complex depositions, which include immunoglobulins and complement proteins. As part of one study, researchers visualized the heart valves of eight patients with APS using immunofluorescence and immunoperoxidase staining. Complement components C1q, C3c, and C4 were present in the affected heart valves, suggesting that the complement system is in part responsible for the effects of APS on cardiac valves [126]. As this study included a relatively small sample size and merely identified complement presence, no definitive causal relationship can be established. Still, if complement deposition contributes to valvular abnormalities, it may also serve as a contributor to rarer cardiac manifestations of APS. For instance, complement-mediated activation of profibrotic factors such as TNF-α could contribute to fibrosis that interrupts the electrical pathways of the heart, leading to arrhythmia. In another clinical investigation, C1q, C3, and C5b-9 immunostaining revealed colocalization with β2GPI in the occluded femoral arteries and the presence of plasma IgG anti-cardiolipin and anti-β2GPI. Treatment with anti-C5 antibodies, eculizumab, and low-molecular-weight heparin inhibited rethrombosis and improved patient outcome with enhanced revascularization post-arterial surgery [127]. Together, these data indicate the pivotal role of complement factors as primary cofactors in triggering chronic inflammation seen with autoimmune diseases. Figure 3 illustrates the roles of complement in autoimmune conditions associated with acute pericarditis and chronic cardiac remodeling and hypertrophy.

## 6. Infectious Diseases, Complement Activation, and Cardiac Consequences

### 6.1. Rheumatic Heart Disease

Several infectious disease processes are known to result in cardiac inflammation and hypertrophy. Chronic rheumatic heart disease (CRHD), caused by the bacteria *Streptococcus pyogenes* (group A strep), comprises one example. *Streptococcus pyogenes* can cause damage to the heart and cardiac valves in particular, due to its cell wall expression of a virulence factor called M protein [128]. The heart contains several proteins that closely resemble M protein, such as myosin, tropomyosin, and vimentin. Subsequent immune responses, such as T-cell reactivity generated against M proteins, ultimately harm the host heart [128,129]. The complement system is highly involved in this response through both the classical and lectin pathways.

A study published in 2020 that focused on the rheumatic heart disease precursor acute rheumatic fever (ARF) found significantly elevated serum levels of IgG3 and C4, which are both involved in the classical pathway, in children with active ARF [130]. This suggests that the complement system is a significant contributor to the initial immune response mounted against *Streptococcus pyogenes*. More notably, it has been found that the complement system remains activated chronically under these conditions, leading to cardiac sequelae. Another study that compared 100 patients with chronic rheumatic heart disease (CRHD) to 99 healthy patients found that serum concentration of lectin pathway-activating mannose-binding lectin (MBL) (ng/mL) appeared at significantly higher levels in the patients with CRHD compared to healthy controls. Interestingly, this same study also found that serum concentration of MBL did not necessarily correlate to the magnitude of cardiac damage or remodeling as visualized by a transthoracic echocardiogram [131]. Still, the elevation of serum MBL concentration in patients with CRHD suggests that the complement system may contribute to the progression and amplification of the disease. One pathway through which this occurs is via MBL-induced activation of cytokine transforming growth factor (TGF)-β1, which in turn activates fibroblasts. Those fibroblasts subsequently undergo proliferation and migration, contributing to tissue hypertrophy [132,133].

Additionally, TGF-β1, in conjunction with IL-6 or IL-4, causes T cell differentiation into phenotypes that release further inflammatory mediators, such as IL-9 and IL-10 [134]. The most common outcome of CRHD is mitral or aortic valve stenosis with subsequent left atrial and ventricular hypertrophy [131]. While this hypertrophy is generally attributed to the increased pressure of working against a stenotic valve, further research should be performed to fully characterize and unravel the role of complement in this presentation.

### 6.2. Infective Endocarditis

Infective endocarditis (IE) describes the process by which bacteria or fungi infect the endocardium, or inner lining, of the heart. IE is reportedly caused by a multitude of organisms, including *Staphylococcus aureus*, *Streptococcus viridians*, and *Candida albicans*, to name a few common culprits [135,136,137]. To infect the heart’s lining and valves, bacteria and fungi must first be present within the bloodstream, which often means that IE follows IV drug use, prosthetic heart valve placement, and dental procedures [137]. While some bacteria preferentially infect previously damaged heart regions, others affect even healthy tissue.

One study examined the relationship between circulating immune complexes, the complement system, and IE. Levels of soluble C5b-9 (sC5b-9), C3d, C3b(Bb)P, and C3a/C3adesArg were measured via enzyme-linked immunosorbent assay (ELISA) studies, comparing 15 IE patients to 14 healthy volunteers. The assay results determined that the classical pathway was preferentially activated, as demonstrated by increased levels of all measured complement activation products except C3b(Bb)P, which is involved in the alternative pathway [138].

A case report published in 2022 studied the relationship between IE and the development of glomerulonephritis in a 27-year-old Hispanic male who first presented with fever and altered mental status. The patient’s blood and urine cultures tested positive for methicillin-sensitive *Staphylococcus aureus* (MSSA), and a transthoracic echocardiogram found vegetative growths on the patient’s mitral valve. Immunohistochemistry of renal biopsy showed granular C3 deposition on the glomerulus, consistent with glomerulonephritis [139]. It is posited that the initial *S. aureus* infection and colonization of the mitral valve encouraged increased serum levels of C3, which then deposited in the kidney. While bacterial infections directly damage cardiac tissues, infective endocarditis can create an impetus for complement activation and increased inflammatory markers that further injure other host tissues. Specifically, activation of various complement pathways converging in the generation of excessive C3 encourages immune complex deposition onto organs, including the heart.

### 6.3. Coxsackie B Virus

Another infectious etiology of cardiac inflammation and remodeling is Coxsackievirus B (CVB), which causes myocarditis [140]. This disease infects cardiac tissues through interactions involving the coxsackievirus-adenovirus receptor (CAR). This receptor can be found on intercalated disks, structures between cardiomyocytes that allow for electrochemical communication and synchronous muscle activity [141]. The myocarditis caused by CVB often subsequently progresses to dilated cardiomyopathy, an eccentric hypertrophy that results in impaired systolic function and ultimately leads to heart failure [140].

Studies have implicated the complement system in this myocarditis. Specifically, pre-clinical models demonstrate that a deficiency in complement receptors 1 and 2 (CR1 and CR2) in CBV infection is associated with increased cardiac inflammation and fibrosis of the pericardium, the fluid-filled sac surrounding the heart [142]. As previously discussed, CR1 and CR2 are regulatory components of the complement system; their function includes clearance of immune complexes and protection of the host organism from excessive inflammation and subsequent damage [43]. The increased degree of myocarditis seen with deficiencies of these two regulators indicates that the complement system holds responsibility for much of the initial inflammatory response in CVB infections. Another study published in 2025 further demonstrated that mice infected with a CVB strain exhibited increased gene expression associated with complement activation in myocardial tissues. This process appeared to be mediated by damage to local mitochondria. Previous research has shown interaction between mitochondria and the complement system, with mitochondrial release of DAMPs triggering complement activation [143]. In this experiment, upon isolation of injured mitochondria and subsequent exposure to human serum, Western blot analyses found significant amounts of C5, C1r, and C4 [144]. This same study discovered through immunohistochemical staining that human tissues infected with the virus also exhibited significantly more staining for C5-9 than healthy controls, further suggesting that complement system activation and MAC deposition give rise to myocardial changes [144].

Furthermore, a study performed using decay-accelerating factor (DAF) synthesized as an IgG1-Fc fusion protein (DAF-Fc) aimed to prevent complement activation by treating mice with this protein early in the infection course. Results indicated that DAF-Fc administration protected the cardiac tissue from infection, therefore decreasing the amount of cell death as visualized by hematoxylin and eosin staining of myocardial sections, as well as semi-quantification of lesion area, calcification, and necrosis, amongst other factors. Viral load in myocardium was measured through plaque assay and was significantly lower in DAF-Fc-treated mice than in controls [145]. Together, these studies indicate that dysregulation of the complement pathway, whether by loss of negative regulators or stimulation of a new activator, contributes to myocardial remodeling in the wake of CBV infection. Future research should determine the avenues through which the complement cascade contributes to myocardial damage and remodeling in response to CVB, as intervention in this mechanism may decrease severity and prevent disease progression.

### 6.4. Chagas Disease

Chagas Disease is caused by a parasite called *Trypanosoma cruzi* and can cause dilated cardiomyopathy [146]. *T. cruzi* is known to infect the heart, causing damage to muscle tissue and even valves, and nearly a third of people infected by this parasite will develop chronic cardiac disease later in life [147,148]. Thus, there is a great need for effective, targeted interventions that can treat *T. cruzi* infections while also preventing inflammatory damage to the heart.

Unique amongst the other infectious causes of cardiomyopathy, *T. cruzi* demonstrates resistance against complement-mediated attacks through various avenues. Research has shown that *T. cruzi* amastigotes, or parasites in their replicating stage, can activate complement but are themselves resistant to complement-mediated destruction due to their expression of a protein structurally similar to DAF [146]. Despite this resistance, terminal complement proteins, including the MAC, deposit on myocardial cell surfaces, leading to heart inflammation. Additionally, studies have shown that *T. cruzi* expresses complement C2 receptor inhibitor trispanning (CRIT) protein, which prevents the generation of C3 convertase. This mechanism further allows the parasite to evade complement-mediated killing [149]. The parasite also damages the vasculature by producing endothelin-1, a potent vasoconstrictor [150]. While vasoconstriction is a normal physiologic process in response to certain stimuli, excessive vasoconstriction may result in ischemia and subsequent endothelial cell infarction. In this case, cells die via necrosis, which creates an inflammatory response via the release of DAMPs [151]. DAMPs, in turn, activate the complement system; the formation of complexes between mannose-binding lectin and DAMPs activates the lectin pathway, and C1 binding IgG or IgM expressed on DAMPs can activate the classical pathway [152]. Altogether, the complement cascade is implicated in the initial response against the *T. cruzi* parasite and in the following tissue damage. Further work should investigate ways in which treatments can combat the parasite’s molecular mimicry and evasion of the complement system.

### 6.5. Septic Cardiomyopathy

Sepsis is broadly defined as an often-lethal organ dysfunction that occurs because of severe infection and subsequent inflammation [153]. Coagulation and complement cascades activate in this inflammatory response, leading to end-organ damage [154]. Septic cardiomyopathy, or cardiac dysfunction in the setting of sepsis, has an incidence of approximately 80% amongst patients with septic shock. This dysfunction includes, but is not limited to, decreased systemic vascular resistance that leads to low blood pressure as well as impaired systolic function and cardiac output [155]. While septic cardiomyopathy is largely reversible upon recovery, patients who experience sepsis have a greater risk for cardiac diseases such as atrial fibrillation, myocardial infarction, and heart failure [156]. Currently, the most common treatments for sepsis include fluid resuscitation, infection source control, antibiotics, supplemental oxygen, and vasopressors [157]. Because sepsis possesses a high mortality rate, roughly estimated at around 40% in hospitals, there is a high demand for research into novel treatments [157], and one potential target for therapy is the complement system.

A study in primate models infected with *Escherichia coli* to mimic the response to sepsis has shown that inhibition of the complement system reduced vascular endothelial damage and correlated with improved cardiac function. The C3 convertase inhibitor compstatin decreased complement product deposition in organs and influenced the coagulation side of the immune response by mitigating thrombosis. Immunofluorescence staining of lung sections demonstrated that primates treated with compstatin had decreased amounts of pro-thrombotic factors (tissue factor and PAI-1) and increased amounts of anticoagulant factors (tissue factor pathway inhibitor (TFPI) and thrombomodulin) [158]. Because sepsis can result in widespread thrombosis, it is essential to note the crosstalk between the coagulation cascade and the complement pathway. Specifically, TFPI can inhibit the lectin pathway by inhibiting MASP-2 [159]. At the same time, endothelial cell expression of complement proteins drives the generation of tissue factor, which is pro-thrombotic [160,161].

Histopathologic examination of several organ tissues (lung, adrenal gland, spleen, liver, and kidney) found that levels of MBL, C3b, and C5b9 deposition were lower in the compstatin treatment group than in controls. While blood pressure drops drastically in sepsis, primates treated with compstatin showed higher mean arterial pressures than the controls, suggesting that complement inhibition in sepsis exerts cardiovascular-protective effects [158].

Previous research has shown that high levels of complement, specifically C5a, are associated with cardiomyocyte dysfunction and impaired cardiac contraction and relaxation [155]. A study on WT mice infected with *Escherichia coli* identified a “myocardial cytokine storm” that impaired cardiac function. In the C5aR double-knockout mice, however, mitigation of this cytokine response protected from myocyte dysfunction. Similarly, WT mice treated intravenously with anti-C5a antibody displayed significantly decreased levels of proinflammatory IL-6, MIP-1α, and MIP-2 in their cardiomyocytes. Results from the study suggest that targeting the complement system prevents downstream release of inflammatory mediators such as cytokines and chemokines, decreasing cardiac inflammation and dysfunction [162]. Additionally, increased levels of C5a in sepsis are implicated in reduced levels of cardiomyocyte Ca^2+^-ATPase (SERCA2) and the Na^+^/Ca^2+^ exchanger (NCX), two membrane transport proteins with vital function in the depolarization-repolarization cycle, and therefore contraction and relaxation, of cardiomyocytes. High C5a thus contributes to cardiac mechanical dysfunction through excessive inflammation and interference with proper electrochemical equilibrium. While a C5a monoclonal antibody (mAb) has previously been tested and found safe for humans, more research should be performed to gauge the cardio-protective role of C5a mAb in the development or prevention of post-sepsis cardiac disease [155].

## 7. Noninfectious Diseases, Complement Activation, and Cardiac Consequences

### 7.1. Obesity and Diabetes Mellitus

Obesity is generally defined by a body mass index (BMI) ≥ 30 kg/m^2^, a distinct classification from overweight, which includes BMIs ≥ 25 kg/m^2^ but less than 30 kg/m^2^. Obesity carries risk factors for a multitude of other diseases, including but not limited to diabetes mellitus type II, cardiovascular disease, sleep disorders, and hypertension [163,164]. An excessive percentage of body fat also induces a low-grade chronic inflammatory state, as adipose tissue itself contains cells involved in the inflammatory response. In obesity, macrophages in adipose tissue shift to a proinflammatory M1 phenotype, increasing other inflammatory mediators such as IL-10, TNF-α, and inducible nitric oxide synthase (iNOS) [165]. In the Diabetes Insulin Glucose Infusion and Myocardial Infarction 2 (DIGAMI 2) clinical trial conducted on 417 patients, 141 type 2 diabetic patients with myocardial infarction exhibited significantly elevated soluble TCC (sC5b-9) plasma levels at the time of admittance (154 (110–219) ng/L versus 129 (95–177) ng/L (*p* = 0.003). Independent of sC5-9, plasma MASP2 levels were also elevated, although not significantly. This suggests the involvement of complement activation and that sC5-9 may serve as a predictive marker during the induction of subsequent myocardial damage and for future cardiovascular events in diabetic patients [166].

In terms of cardiac-specific consequences, obesity contributes to adverse remodeling of the heart. Preclinical models show an association between obesity and decreased conduction velocity and an increase in atrial fibrillation, whether spontaneous or induced, showing disturbances to the electrical pathways of the heart [167]. Additionally, several post-mortem studies on hearts in those with obesity identified a positive association between obesity and left ventricular mass [168,169]. Excessive fat can also accumulate in cardiomyocytes, a phenomenon termed cardiac lipotoxicity [169]. Interestingly, complement pathway function in obesity-induced cardiac remodeling is attributed to cardiac fibroblast activity. Cardiac fibroblast expression of a receptor called protease-activated receptor 2 (PAR2) is associated with decreased cardiac expression of classical complement factors C1R, C1QL1, C4b, and complement factor D. It corresponds to increased expression of CD55, a negative regulator of the alternative complement cascade, which indicates the existence of a compensatory mechanism to limit inflammation. PAR2 also upregulates cyclooxygenase 2 and proinflammatory cytokines such as IL-8 and IL-8 receptor, IL-1, and TNF-9 [170]. Researchers posit that PAR2 has both pro- and anti-inflammatory roles that can vary with comorbid conditions. Thus, these data suggest that targeting cellular receptors such as PAR2 could potentially prevent adverse cardiac remodeling by the alternative complement pathway and prevent cardiac failure-related mortality in patients with obesity.

### 7.2. Dyslipidemias

Dyslipidemia is a collective term that refers to disorders of lipid metabolism as well as abnormal levels of lipids in the blood, including but not limited to high-density lipoprotein (HDL), low-density lipoprotein (LDL), and total triglycerides [171,172]. Many conditions fall under the classification of dyslipidemia, such as hyperchylomicronemia, hypercholesterolemia, and dysbetalipoproteinemia, also known as hyperlipoproteinemia, to name a few. Dyslipidemias occur through both genetic and environmental means, and their presence is most feared due to their involvement in the pathogenesis of atherosclerotic cardiovascular disease (ASCVD). High lipid levels physically occlude arteries but also contribute to the generation of reactive oxygen species (ROS), which contribute to oxidative stress and subsequent endothelial damage [172,173]. Higher LDL and triglycerides are also associated with increased left ventricular end-diastolic volume (LVEDV) and left ventricular mass, respectively [174]. Additionally, LDL is positively correlated with levels of plasma C3 [175]. The relationship between lipids and the complement system is responsible for atherosclerosis and may influence subsequent cardiac remodeling.

In the pathogenesis of atherosclerosis, cholesterol deposits form in the subendothelial space of a blood vessel. Upon being oxidized, this cholesterol releases DAMPs that trigger an inflammatory response, including the activation of immunoglobulins, C-reactive protein, complement C1q and mannose-binding lectin, and factor H. The presence of deposited cholesterol also encourages nearby endothelial cells to release inflammatory markers, especially C3a and C5a, which further potentiates inflammatory damage to the blood vessel [176]. Ultimately, this results in greater plaque formation and vascular smooth muscle hyperplasia, which contribute to atherosclerosis [177]. It is important to note that the complement system has also been implicated in plaque erosion, or the loss of the endothelial lining over the cholesterol plaque, which can also lead to subsequent cardiovascular events such as thrombosis and infarction [178].

In the coronary arteries, the development of a cholesterol plaque causes blood vessel narrowing. If the plaque ruptures, a thrombus quickly forms, which may completely cut off blood supply to regions of the myocardium and cause a myocardial infarction [179]. At least 70% of acute myocardial infarctions are caused by the rupture of an atherosclerotic plaque [180]. In the aftermath of a myocardial infarction, serum levels of anaphylatoxins C3a and C5a are significantly increased after 16 h. In post-mortem examinations of 12 patients, complement proteins C3d, C4d, and C5b-C9 were present in damaged, ischemic tissue [181,182,183]. This is attributed to the chemotactic properties of C3a and C5a, as both anaphylatoxins stimulate human immune cell migration. Upon damage to cardiac tissue, C3a and C5a upregulation facilitate mast cell infiltration of the affected area. Mast cell degranulation occurs, releasing histamine, tryptase, and chymase; subsequently, tryptase and chymase cause C3 activation, contributing to complement deposition in the affected myocardium [184,185]. Thus, it appears that the complement system can inadvertently promote plaque formation and is also involved in the response to plaque rupture, myocardial infarction, and subsequent tissue remodeling.

### 7.3. Systemic Hypertension

Systemic hypertension is defined by a systolic blood pressure over 130 mmHg and a diastolic blood pressure over 80 mmHg. It has been estimated that approximately 1.39 billion adults worldwide live with hypertension, making it a widely prevalent disease [186]. Hypertension may be idiopathic, termed primary or essential hypertension, or secondary and caused by mechanisms such as genetics, renal artery stenosis, and sleep apnea, to name a few [187,188,189]. Secondary hypertension is also commonly caused by imbalances in the hormones of the renin–angiotensin–aldosterone system (RAAS) [1]. Elevated systemic blood pressure creates an increased afterload, or force, that the heart must oppose and overcome when pumping blood through the rest of the body. Chronic hypertension can thus induce cardiac remodeling; specifically, the left ventricle gains mass through hypertrophy and fibrosis to overcome an increased afterload [190]. Of interest in this review is the role of complement in the relationship between hypertension and cardiac remodeling.

The complement system has previously been linked to the development of hypertension itself. One longitudinal cohort study on 2178 healthy men ages 35 to 50 reported that high serum C3 levels were predictive of later development of hypertension [191,192]. Additionally, renin has been reported to be a mediator of the RAAS and an enzyme capable of cleaving C3 into C3a and C3b [193]. In some instances of hypertension linked to high renin, such as renal artery stenosis, there could consequently be higher serum levels of serum complement, which contribute to adverse cardiac outcomes [194].

Angiotensin II (ATII), another RAAS member, increases blood pressure by upregulating aldosterone. This hormone acts on the kidneys to help the body retain sodium and fluid. ATII is often elevated in secondary hypertension, and drug treatment of hypertension frequently involves either preventing its synthesis or blocking its receptor [195]. In a preclinical study, an experimental group of WT mice received ATII injections to observe the association between hypertension and complement activation. After injection, the mouse sera contained significantly increased levels of C3a and C5a. The mouse hearts themselves, examined via quantitative reverse transcriptase polymerase chain reaction (qRT-PCR), demonstrated higher expression of C3a receptor (C3aR) and C5a receptor (C5aR), with a much greater increase in C5aR compared to C3aR [10]. As part of the same study, WT mice received an anti-mouse C5 monoclonal antibody before any injection of ATII; the hearts of these mice had less fibrosis and remodeling compared to those without the antibody treatment, as visualized by immunohistochemical staining, quantifying α-smooth muscle actin (α-SMA), and through PCR measurement of α-SMA, collagen I, and collagen III mRNA levels [10]. C3a and C5a interact with fibroblasts, encouraging their differentiation, and contribute to extracellular matrix synthesis, implicating these anaphylatoxins in tissue remodeling [196]. These findings indicate that anaphylatoxin inhibition may be the key to preventing cardiac hypertrophy in cases of hypertension.

### 7.4. Pulmonary Arterial Hypertension

Pulmonary hypertension (PH) is less prevalent than systemic hypertension. Still, it has a more direct cardiac impact, closely linked to right ventricular hypertrophy and right heart failure due to increased pressure in the pulmonary circuit [197,198]. Pulmonary hypertension is characterized by a mean pulmonary arterial pressure greater than 20 mmHg [199]. There are five classifications of PH: (1) idiopathic or genetic PH, also known as pulmonary arterial hypertension (PAH), (2) PH due to left heart disease, (3) PH due to pulmonary disease, (4) PH due to chronic thromboembolism, and (5) PH of unknown origin [200]. Patients with PH present with symptoms of right heart dysfunction, such as excessive dyspnea and lower extremity edema [198]. Right ventricular remodeling is an adaptive response to chronic high pulmonary arterial pressures, or a high afterload. This response, however, becomes maladaptive when hypertrophy leads to a decline in diastolic or systolic function [201].

**Figure 4:**
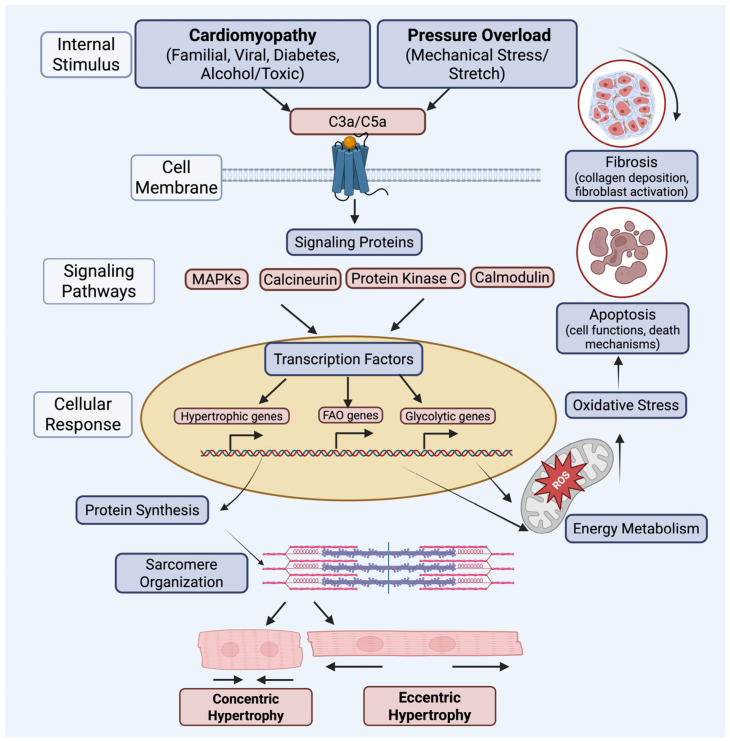
Role of complement in metabolic syndrome-related cardiac hypertrophy. Complement anaphylatoxins, C3a and C5a, contribute to metabolic syndrome-related cardiac hypertrophy through their involvement in inflammation and effects on cardiac cells. C3a and C5a receptors are expressed in the heart. Activation of these receptors can trigger downstream mediators signaling pathways, including MAPKs, protein kinases, and calcium-dependent cyclic AMP (cAMP)-regulating proteins calmodulin and the phosphatase calneurin, which lead to transcriptional changes. Changes in transcription of hypertrophy-related genes, fatty acid oxidation (FAO) genes, and glycolytic pathway genes can result in metabolic dysfunction in the cardiomyocytes and potentially contribute to hypertrophy. Concentric hypertrophy involves an increase in heart muscle thickness without a change in the overall size of the heart chamber, while eccentric hypertrophy involves an increase in both muscle thickness and chamber size. Mechanical stretch due to pressure or volume overload also leads to anaphylatoxin receptor signaling, in addition to activating myofibroblast activation, resulting in cardiac fibrosis. Prolonged pressure overload and sustained inflammation lead to maladaptive remodeling, cell death, and cardiac failure.

The complement system is known to cause vascular remodeling in PAH, but whether complement is involved in the cardiac hypertrophic response remains to be seen [15]. That said, plasma levels of complement proteins, especially those involved in the alternative and terminal pathways, correlate with PAH patient outcomes [202]. Other data suggest that plasma levels of complement factor H and factor D can be part of a prognostic measure in PAH [203]. In a recent preclinical study, rats were given monocrotaline to induce PAH, and an upregulation in plasma C3 was observed. The experimental group received CP40-KK, an analog of the complement C3 inhibitor CP40, which inhibited C3 cleavage and was also found to block NF-κB phosphorylation and NLRP3 inflammasome activation. Downstream effects included reduced release of other proinflammatory mediators, primarily interleukins. This led to decreased vascular remodeling and better survival rates [204]. A different study further corroborates the importance of C3 in the pathogenesis of PAH-induced right heart failure. Here, right ventricular failure was induced in mice via pulmonary artery constriction (PAC), with C3d found in the right ventricle only after this treatment. The same researchers then examined the effects of PAC on both wild-type and C3 double-knockout mice. They found that the C3 double-knockouts exhibited less right ventricular remodeling and fibrosis and less right ventricular systolic dysfunction. The experiment was repeated with complement factor D (CFD) double-knockout mice. It yielded the same results, with the CFD double-knockout mice showing better correct ventricular function and less fibrosis and remodeling [31]. These results indicate the significant role of C3 in right ventricular remodeling and subsequent right heart failure in PAH. Figure 4 illustrates the molecular mechanisms of anaphylatoxin signaling and pathogenic consequences in metabolic disorders, which lead to cardiac hypertrophy.

### 7.5. Myocardial Infarction, Ischemia, and Reperfusion

Activation of the complement system in response to myocardial infarction (MI) has been implicated in both the process of infarction and ischemia, as well as the subsequent fibrosis and remodeling of cardiac tissue. Results from rabbit models suggest that complement proteins are synthesized locally from the heart in settings of infarction; specifically, C1q had an mRNA expression 20 times greater in infarcted tissue compared to the normal heart in rabbits modeling ischemia and reperfusion [183]. Depositions of C4d, C3d, and C5b-9 of the membrane attack complex were also found on infarcted tissue in both the acute and, notably, the chronic state [183]. This data indicates that complement proteins contribute to myocardial damage acutely in the setting of an MI and may additionally have long-lasting effects in the aftermath by continually facilitating inflammation and cardiomyocyte damage.

In considering the long-term effects of complement activation on myocardium, it is important to evaluate the relationship between complement levels and overall patient outcomes in the aftermath of a myocardial infarction. A study on a population of 864 ST-elevation myocardial infarction (STEMI) patients who underwent percutaneous coronary intervention (PCI) discovered that patients with a left ventricular ejection fraction of less than 40% also had higher plasma levels of terminal complement complex (or membrane attack complex) compared to patients with a higher ejection fraction [205]. As ejection fraction is a measure of cardiac function, it is notable that increased levels of complement activation correlate with worse cardiac outcomes amongst this population of STEMI patients. Additionally, a different study performed on 16 STEMI patients who underwent PCI where half of the study population experienced a subsequent microvascular obstruction (MVO). It found that patients with an MVO had gene ontology enrichment analyses consistent with increased complement and coagulation cascade protein expressions [206]. However, the specific proteins and genes upregulated were not mentioned. As the study was small in scope, there is a clear need to further explore how complement activation can impair cardiac functioning and even contribute to other pathologic processes in the wake of a myocardial infarction and subsequent reperfusion therapy.

Studies have been conducted to determine whether the inhibition of complement activation may reduce the size of myocardial infarction. One experiment performed on mice administered with C1 inhibitor (C1INH) found reduced infarct size in the treated mice compared to untreated controls after occlusion of the left anterior descending branch of their coronary arteries and subsequent perfusion [207]. Additionally, levels of polymorphonuclear cells, which are posited to be the greatest contributor to inflammation and damage in myocardial infarctions, were significantly decreased in mice that received the C1 inhibitor treatment [207]. Altogether, these results suggest that the inhibition of complement activation may exert a downstream effect of preventing the activation and accumulation of polymorphonuclear cells such as neutrophils which contribute to the inflammatory response and subsequent myocyte damage. Alternatively, the authors also state that this downregulatory effect may not be entirely from C1INH’s protease inhibitory role alone. They posit that results could also be due to its interference in the interactions between endothelial cell surface selectins and their ligands on leukocytes, ultimately downregulating polymorphonuclear cell recruitment [207]. While inhibition of complement appears to be a promising avenue to consider exploring in the acute treatment of myocardial infarction, further research must be performed to characterize the pathways which potential complement-inhibiting therapeutics would modulate and their long-term effects on cardiac outcomes.

### 7.6. Crosstalk Between Coagulation Cascade and Complement in Cardiovascular Events

As discussed in the infectious endocarditis and septic cardiomyopathy, the mechanisms of the coagulation cascade and complement activation processes are deeply interconnected. A reciprocal relationship between the two defense systems influences the pathogenesis of cardiomyopathy by promoting myocardial injury, inflammation, and fibrosis. For instance, the key proteases of the coagulation cascades, Factor IIa (also known as thrombin), Factor Xa, and Factor Xia directly cleave and activate complement factors C3 and C5. In the absence of C3, thrombin directly cleaves C5 into C5a to initiate an inflammatory response and MAC-mediated cell lysis [208]. Parallelly, platelet activation also enhances complement activation by secreting complement-stabilizing factors such as chondroitin sulfate and CFD, and microvesicular contents, including complement components, promote both cascade activities [209,210,211]. In patients with end-stage arrhythmogenic right ventricular cardiomyopathy (ARVC), an inherited cardiomyopathy that presented with fibrofatty replacement of cardiac tissues, complement activation was evident [212]. Both ventricles of these patients demonstrated increased expressions of C3, C6, C7, C8, and C9, and C5aR1 was observed by deep proteome analysis. The plasma samples revealed significantly increased levels of sC5b9 complex and thrombin, suggesting a crosstalk between the two cascades leading to all-cause mortality [212,213]. In a mouse model of ARVC, in which desmin deficiency recapitulates the pathology of human ARVC, it was demonstrated that coagulation cascade activation as an early event exacerbated complement activation. Inhibition of thrombin function by lepirudin significantly reduced the myocardial injury [75]. Interestingly, in desmin/C3 double knockout mouse strains, significantly low thrombin levels corroborated reduced cardiac pathology, implicating it in the pathophysiology by activating the complement cascades.

## 8. Treatments and Clinical Trials for Cardiac Inflammation and Hypertrophy

There is currently little research on therapeutics that target complement, as it functions in atherosclerosis and mediates cardiac hypertrophy or damage. Recent and ongoing clinical trials slot into two main categories: (1) studies that examine the utility of complement antagonists in individuals at increased risk of complement-mediated cardiac damage and (2) those evaluating drugs that operate on pathways influenced by or influencing complement activity.

Three seminal trials tested the effectiveness of pexelizumab, a humanized monoclonal antibody fragment that binds to C5 to block terminal complement activation, in patients receiving cardiac surgeries. Existing work suggests the role of complement in post-cardiopulmonary bypass inflammatory responses that lead to complications such as organ dysfunction, bleeding, and thromboembolism [214]. One study compared inflammatory markers between patients who received coronary artery bypass grafting (CABG) with and without cardiopulmonary bypass (CPB). While both groups demonstrated subsequent increases in complement proteins C3a and C5a, IL-8 and neutrophil elastase levels were markedly higher in the CPB sample. These patients also exhibited increased neutrophil, monocyte, and white blood cell counts [215]. In this context, the first two trials examined the ability of pexelizumab to reduce complement-mediated complications such as chest pain, myocardial infarction, and heart failure after CPB (Study 1, Study 2, Table 1). Published results describe a significant reduction in the rate of myocardial infarction and/or death through postoperative day 30 in those who received pexelizumab. Incidence of these complications decreased by 28% in individuals with two pre-specified cardiovascular risk factors and 44% in those with three or more risk factors [216]. The third clinical trial looked at the role of pexelizumab in reducing 30-day mortality in patients undergoing reperfusion therapy for myocardial infarction (Study 3, Table 1). In this group, researchers found no significant difference in mortality between those treated with percutaneous coronary intervention (PCI) alone versus with both PCI and pexelizumab for acute ST-elevation myocardial infarction (STEMI). Composite end points of death, cardiogenic shock, and congestive heart failure through 30 and 90 days also occurred at similar rates between the two treatment groups [217]. Although the trial of pexelizumab in participants who received PCI for MI failed to show similar results as the study examining its therapeutic potential in patients undergoing CPB, discrepancies could derive from more inconsistent patterns of ischemic-reperfusion injury in MI. Cardiopulmonary bypass redirects blood flow away from the heart during surgery to facilitate direct manipulation of cardiac tissue and vasculature without devastating hemorrhage, which causes a short-term global ischemia. Myocardial infarction, in contrast, presents quite heterogeneously depending on the specific vascular territory impacted and the time from symptom onset to medical or surgical intervention. Although gold standard treatment guidelines in the United States recommend PCI initiation within 120 min of symptom onset and within 90 min of entering the hospital, individuals in practice may receive interventions outside of this ideal timeframe. It is possible that complement inhibitors such as pexelizumab may function more effectively to impact mortality in participants who present with MIs which involve larger regions of cardiac tissue or who suffer longer periods of cardiac ischemia prior to reperfusion. Further studies could examine the utility of pexelizumab in these sub-populations of individuals with MIs.

Avacopan is a complement C5a receptor (C5aR) antagonist that received FDA approval in 2021 for treating anti-neutrophil cytoplasmic antibody (ANCA)-associated vasculitis. The mechanism of injury in this autoimmune disease involves ANCA-mediated excessive activation of neutrophils in small vessels with subsequent release of inflammatory cytokines, production of reactive oxygen species, and formation of neutrophil extracellular traps (NETs) [218]. Complement activation contributes to and is amplified by this process, as the binding of anaphylatoxins C3a and C5a with their respective receptors similarly encourages neutrophil chemotaxis and NET formation [219]. A 2020 clinical trial examined the effect of avacopan at both regular and supratherapeutic doses on the heart rate-corrected QT interval (QTc) (Study 4, Table 1). QT interval length correlates positively with the risk of cardiac arrhythmias, but this study found no significant effect of avacopan on participants’ QT/QTc intervals [220]. Given that cardiac hypertrophy similarly predisposes individuals to arrhythmias, any significant lengthening of the QT interval in study participants would represent a possible contraindication for future use on patients with complement-mediated cardiac damage and hypertrophy. The lack of such findings leaves an avenue open for future investigation of avacopan as a therapeutic option in other forms of cardiac and vascular damage, which are defined by significant contributions from complement and neutrophil activation. Another complement antagonist, soluble complement receptor 1 (TP10), accelerates the decay of C3 and C5 convertases to reduce terminal complement activation. Researchers investigated its utility in reducing the incidence of death or MI in women receiving high-risk cardiac surgery and cardiopulmonary bypass (Study 5, Table 1). While previous work demonstrated a marked 36% decrease in death and MI in male participants with similar participation criteria, this study showed no significant effect on death and MI incidence in the 297 women examined [221]. Of note, researchers opted to exclude patients with a history of MI within 72 h before their surgeries, so it remains unclear if TP10 would exert a more marked effect on populations with established ischemic injuries [222]. Results also raise questions regarding whether gender-specific differences in complement activation and cascade amplification during cardiac surgery exist and possibly cause significant discrepancies in treatment efficacy.

IL-6 is a pleiotropic cytokine released in response to tissue damage or infection, with roles in both innate and adaptive immune responses. Following its production, IL-6 encourages lymphocyte migration and activates B- and T-cell responses while also triggering the release of additional inflammatory mediators. Despite not directly binding to complement, IL-6 inhibitor tocilizumab has recently been shown to decrease activity of both the classical and alternative complement pathways in patients with rheumatoid arthritis (RA) [223]. These results follow previous observations of its ability to reduce acute-phase reactants like C3 and C4 and cause hypocomplementemia in individuals receiving treatment [224]. Furthermore, IL-6 contributes to cardiac disease specifically by promoting the development and destabilization of atherosclerotic plaques [225]. Given the increased presence of cardiac risk factors and atherosclerosis in patients with RA, a phase 4 trial sought to evaluate the effect of tocilizumab on atherosclerotic plaque formation in this population [226]. It unfortunately failed to garner sufficient enrollment for completion but represents an intriguing avenue for further investigation given that overall RA prevalence estimates reach as high as 1% (Study 6, Table 1). A phase 4 trial evaluating the effect of tocilizumab on atherosclerotic plaque formation in RA patients failed to garner sufficient enrollment for completion. Still, it represents an avenue for further investigation (Study 6, Table 1). Another study examined the effectiveness of p38 kinase inhibitor BMS-582949 in reducing arterial inflammation in 72 participants with atherosclerosis (Study 7, Table 1). Complement protein C5a can induce p38 mitogen-activated protein kinase (MAPK), crucial in cellular responses to stress. Previous studies have demonstrated the protective effect of p38 MAPK inhibitors in murine models of lupus nephritis and endotoxin-induced inflammation, processes associated with or partly caused by increased complement activity [227,228]. Although published results of this trial show no significant difference in inflammation between the placebo and treatment groups, other studies indicate possible benefits of p38 kinase inhibitor use [229]. Additionally, a 2022 study of murine cardiac function and hypertrophy noted a history of poor results in clinical trials focused on inhibition of individual members of the p38 family, which informed the decision to examine p38 activator proteins MKK3 and MKK6 [230]. Future studies of the utility of p38 inhibition in attenuating cardiac damage may consider focusing on inhibition of a broader spectrum of p38 family members, as BMS-582949 binds specifically to p38α.

Research into therapeutics that target pathways parallel to complement activation or directly influenced by it also presents promising avenues for treatment. A set of phase 2 studies previously assessed the utility of 5-lipoxygenase (5-LO) inhibitor VIA-2291 in vascular inflammation in patients with acute coronary syndrome or those undergoing elective carotid endarterectomy (Study 8, Study 9, Table 1). The enzyme 5-LO contributes heavily to leukotriene production, and leukotrienes mediate inflammation in both atherosclerosis and ischemic injuries [231]. Anaphylatoxin C5a similarly stimulates the production of leukotrienes during inflammatory responses [232]. Results of the trial on patients with acute coronary syndrome showed a marked decrease in leukotriene levels but no significant reduction in vascular inflammation [233]. The trial examining individuals undergoing elective carotid endarterectomy yielded similar results, with a statistically significant decrease in serum and urine leukotrienes in the treatment population but no significant difference in the primary outcome measure of percent cross-sectional area of macrophages in plaque tissue. One possible limitation of this study concerns the 12-week timeline for treatment. Although a significant portion of macrophages present in atherosclerotic plaques are monocyte-derived and commonly recruited to vascular tissues as inflammation progresses, a population of self-replenishing long-lived resident macrophages also inhabits the vasculature. These largely adventitia-residing macrophages typically exert local homeostatic functions and contribute to or ameliorate inflammation as needed, but it is also possible that they may actively contribute to atherosclerotic progression in conditions that cause widespread macrophage dysregulation [234]. Without comprehensive quantification of their contribution to and presence in local inflammatory processes and atherosclerotic plaque development under a variety of conditions, it is difficult to determine whether the original trial’s lack of reduction in plaque macrophage population truly represents a lack of effectiveness of VIA-2291 in reduction in macrophage populations or if the population in question has a lifespan longer than that of the treatment duration. Additionally, the classic dichotomy of the M1 proinflammatory versus M2 anti-inflammatory macrophage phenotypes may also experience disruption in the context of complement dysregulation [235]. A 2021 study of complement and macrophage crosstalk in human lupus nephritis crescent formation found widespread co-localization of complement receptor C3aR and M2 macrophage marker CD163^+^ in renal biopsies of participants. These M2 macrophages were the dominant subpopulation and correlated strongly with complement activation and crescent formation [236].

Another trial observed the rates of cardiovascular events in patients with coronary artery disease treated either with darapladib or a placebo (Study 10, Table 1). Darapladib is a lipoprotein-associated phospholipase A2 (Lp-PLA2) inhibitor that stabilizes atherosclerotic plaques by regulating vascular inflammation mediated by Lp-PLA2. Although Lp-PLA2 does not directly affect the complement system, Lp-PLA2 can hydrolyze oxidized LDL to produce inflammatory lysophosphatidylcholine and oxidized nonesterified fatty acids. This Lp-PLA2-digested LDL can then activate complement [237]. Upon follow-up, the primary outcome of significant events such as nonfatal MI, cardiovascular mortality, and nonfatal stroke did not differ significantly between placebo and Darapladib-treated groups. Decreases in composite secondary outcomes like MI, mortality, and urgent coronary revascularization, however, suggest a possible role for darapladib in more populations with certain risk factors (https://www.acc.org/latest-in-cardiology/clinical-trials/2014/09/01/16/30/stability (accessed on 15 September 2025)).

A clinical trial seeking to test the utility of MK-0736, a selective 11β-hydroxysteroid dehydrogenase type 1 (11β-HSD1) inhibitor, in reducing macrophage content of atherosclerotic plaques was terminated due to lack of recruitment (Study 11, Table 1). The enzyme 11β-HSD1 converts cortisone to cortisol within cells and is sensitive to upregulation by inflammatory cytokines such as TNF-α and IL-1β [238,239]. These same cytokines can induce or directly result from complement activation.

While most current trials assess new therapeutics, a few seek to further clarify the contribution of complement to cardiac damage. An observational study completed in 2018 examined the effect of genetic variations in complement on cardiovascular risk in adolescents (Study 12, Table 1). It identified a correlation between increased C3 and C4 levels and worsened endothelial function, with elevated C4 also corresponding positively with inflammatory markers and negatively with high-density lipoprotein (HDL). Additionally, researchers identified a significant relationship between gene copy numbers of all four C4 subtypes and HDL levels [240]. A different study with an unknown completion status examined the presence and possible role of the lectin pathway in patients resuscitated after out-of-hospital cardiac arrests. Variables measured included concentrations of plasma lectin pathway proteins on three consecutive days following cardiac arrest, and researchers also sought to clarify the relationship between these lectin pathway protein concentrations and mortality (Study 13, Table 1).

In light of the above discussions, it is noteworthy that biologics targeting complement activity have been tested as therapeutics for refractory autoimmune and infectious diseases. A recent study demonstrated the efficacy of eculizumab, a potent C5 inhibitor, in treating antiphospholipid syndrome-related multiple thrombosis and pulmonary embolism. The patient was refractory to thrombolytics (enoxaparin, fondaparinux, apixaban, rivaroxaban, and warfarin), antiplatelet drugs including aspirin, and adjunctive therapies such as hydroxyquinoline and steroids. However, oral administration of eculizumab along with the refractive therapeutic regimen improved APS presentation without the recurrence of thrombosis [241]. Eculizumab is also approved for use during pregnancy in the event of pediatric catastrophic APS (CAPS), which is prevalent in 1% of obstetric APS patients. It can treat conditions such as atherosclerosis and cardiac valve disease to reduce the risk of premature birth or fetal death [242]. Although not directly associated with intervening cardiac events, the C5a anaphylatoxin inhibitor, vilobelimab, is another alternative pathway inhibitor that has been approved for the treatment of acute cardiopulmonary arrest in critically ill COVID-19 patients and was considered safe for mechanically ventilated patients (ClinicalTrials.gov: NCT04333420) [243]. A randomized clinical trial was also conducted in COVID-19 patients to investigate the efficacy of the C5aR1 inhibitor avdoralimab (ClinicalTrials.gov: NCT04371367). The report suggested that avdoralimab did not improve the WHO clinical scale score on days 14 and 28 of the administration [244]. The C3 inhibitor AMY-101 from Amyndas Pharmaceuticals is another example of a complement biologic that was recently tested in gingivitis and periodontitis patients with a significant risk of developing cardiovascular diseases (ClinicalTrials.gov: NCT0394444). AMY-101 is labeled as a compstatin that inhibits at the level of C3, which is the converging point of the three complement cascades and therefore inhibits the activation of the downstream pathways [245,246]. MASP2 inhibitor narsoplimab is under review by the FDA, and the decision is expected to be made soon (ClinicalTrials.gov: NCT02222545). Narsoplimab is designed to prevent complement-influenced inflammation and endothelial damage observed in patients with hematopoietic stem cell transplant (HSCT)-associated thrombotic microangiopathy (TA-TMA). The preliminary phase 2 clinical trials showed improved survival and reduced risk of death with multiple comorbidities [247]. Thus, these approaches have ascertained the feasibility of therapeutic targeting of complement components to mitigate inflammation, thereby preventing cardiovascular complications.

**Table 1:** Clinical case studies related to cardiac adversities and their interventions.

No.	Study Title	Conditions	Interventions	Clinical Trial Number	Status
1	Pexelizumab in patients undergoing coronary artery bypass grafting with cardiopulmonary bypass (PRIMO-CABG II)	Coronary Artery Bypass	Pexelizumab	NCT00088179	Completed [215]
2	Effect of Pexelizumab on all-cause mortality and myocardial Infarction in patients undergoing coronary artery bypass graft surgery with cardiopulmonary bypass	Coronary Artery Bypass	Pexelizumab	NCT00048308	Completed [216]
3	Pexelizumab in conjunction with Angioplasty in acute myocardial infarction (APEX-AMI)	Myocardial infarction	Pexelizumab	NCT00091637	Completed [217]
4	A study to assess the effect of Avacopan at therapeutic and Supratherapeutic doses on the QT/QTc interval in healthy participants	NA	Avacopan	NCT05988034	Completed [220]
5	Safety and Efficacy of TP-10, a complement inhibitor, in adult women undergoing cardiopulmonary bypass surgery	Cardiopulmonary Bypass Surgery	TP-10	NCT00082121	Completed [221,222]
6	Effect of subcutaneous ACTEMRA on inflamed atherosclerotic plaques in patients with rheumatoid arthritis	RA, Atherosclerosis	Tocilizumab	NCT02659150	Terminated [223,224]
7	Efficacy study of p38 kinase inhibitor to treat patients with atherosclerosis	Atherosclerosis	BMS-582949	NCT00570752	Completed [227,228]
8	Phase 2 study in vascular inflammation on patients after an acute coronary syndrome event	Acute coronary syndrome	VIA-2291	NCT00552188	Completed [233]
9	Effect of VIA-2291 on atherosclerotic vascular inflammation in patients undergoing elective carotid endarterectomy	Carotid stenosis, Atherosclerosis	VIA-2291	NCT00352417	Completed [233]
10	The stabilization of atherosclerotic plaque by initiation of Darapladib	Atherosclerosis, CAD	Darapladib	NCT00799903	Completed [237]
11	A study of how MK-0736 affects arterial plaque	Atherosclerosis, PAD	MK-0736	NCT00679055	Terminated [238]
12	Complement and cardiovascular risk in adolescents (CCRIA)	Cardiovascular Risk	NA	NCT02821104	Completed [239,240]
13	The complement lectin pathway after cardiac arrest	Cardiac Arrest	NA	NCT02826057	Unknown [240]
14	Vilobelimab therapy for invasively mechanically ventilated patients with COVID-19 (PANAMO)	Critical COVID-19	Vilobelimab	NCT04333420	Completed [243]
15	Efficacy of the C5aR1 inhibitor avdoralimab (FORCE)	Critical COVID-19	Avdoralimab	NCT04371367	Completed [244]

## 9. General Conclusions and Future Perspectives

Although a promising avenue for treating pathologies ranging from autoimmune conditions to infectious diseases, as well as immune system activation during major surgery, complement therapeutics remain constrained by several limitations in development and utilization. Given the immense complexity of the complement cascade, which consists of over 60 proteins, current understanding of the function of individual components largely informs and circumscribes research into novel drugs. A lack of literature elucidating precise signaling mechanisms limits the total number of potential targets for treatment, which is reflected in existing trends in development. Current FDA-approved complement inhibitors either interact with only a few upstream components, such as C3, factor B, and factor D, or bind C5 to block terminal complement activation [44]. This makes sense in the context of known cascade convergence points and the risk of creating medications with redundant function, so future work should examine less well-known culprits. For example, the protein C4a is a recognized anaphylatoxin, but the lack of identification of any proven universal receptor prevents intervention in its signaling pathway [248,249].

In addition, the versatility of complement and its changing activities in varying contexts complicate attempts to achieve desired therapeutic effects via inhibition. For example, the same C3aR deficiency that can improve the clinical picture in murine models of IgA nephropathy can worsen disease presentation in murine models of lupus [250]. Existing research also focuses largely on extracellular complement, but targeting intracellular signaling may generate novel effects. In a similar vein, it remains difficult to develop treatments for medical conditions unless the role of complement in that disease process is validated clinically [251]. Many successful FDA-approved therapeutics, such as C5 inhibitors eculizumab and crovalimab or C3 inhibitor pegcetacoplan, treat paroxysmal nocturnal hemoglobinuria (PNH), a condition with clearly described complement-mediated lysis of red blood cells. Identifying exact complement contribution in other illnesses with more multifactorial contributions to the disease process remains difficult, and any targeted therapeutics may function best in context with other agents.

Lastly, interference with any significant portion of the complement cascade interferes with innate immune system function and runs the risk of increasing susceptibility to infectious disease. Protein C3b is an opsonin that helps mediate the marking of pathogens for phagocytosis and chemotaxis of immune cells. Interference with C3 convertase function or inhibition of C3 theoretically results in reduced opsonization and increased susceptibility to bacterial infection. The C5 inhibitor eculizumab is associated with a higher incidence of Neisseria species infections due to reduced membrane attack complex activity from terminal complement inhibition [250].

In conclusion, the complement system, a key feature of the immune response, is implicated in the pathogenesis of cardiac inflammation and hypertrophy due to various etiologies such as dysregulation of complement, genetic polymorphisms in complement genes, autoimmune diseases, infectious diseases, and non-communicable diseases. This review discusses mechanisms by which the classical, alternative, and lectin pathways contribute to the development of cardiovascular disease. Besides the neutralizing antibodies, modification in the expression or binding of complement regulators, receptors, and other soluble complement proteins would potentially present an opportunity to prevent adverse cardiac outcomes. Therefore, further research and clinical exploration are necessary to determine whether targeting the complement system presents a feasible, cost-effective way of preventing cardiac damage and reducing overall rates of cardiac-related mortality.
